# Gut microbiota signatures associated with lithium treatment and clinical response in patients with bipolar disorder

**DOI:** 10.3389/fmicb.2026.1839847

**Published:** 2026-06-29

**Authors:** Vanessa Palmas, Lívia Cavalcanti de Morais Gama, Suzana Eiko Sato Guima, Giuseppina Sanna, Claudia Pisanu, Cristina Piras, Martina Spada, Anna Meloni, Donatella Congiu, Giovanni Severino, Raffaella Ardau, Caterina Chillotti, Martina Contu, Pasquale Paribello, Marco Pinna, Federico Suprani, Maria Del Zompo, Bernardo Carpiniello, Mirko Manchia, João Carlos Setubal, Luigi Atzori, Alessio Squassina, Aldo Manzin

**Affiliations:** 1 Department of Biomedical Sciences, Microbiology and Virology Unit, University of Cagliari, Monserrato, Italy; 2 Department of Biochemistry, Institute of Chemistry, University of São Paulo (USP), São Paulo, Brazil; 3 Department of Biomedical Sciences, Section of Neuroscience and Clinical Pharmacology, University of Cagliari, Cagliari, Italy; 4 Section of Pathology, Department of Biomedical Sciences, University of Cagliari, Cagliari, Italy; 5 Unit of Clinical Pharmacology, University Hospital Agency of Cagliari, Cagliari, Italy; 6 Section of Psychiatry, Department of Medical Sciences and Public Health, University of Cagliari, Cagliari, Italy; 7 Unit of Clinical Psychiatry, University Hospital Agency of Cagliari, Cagliari, Italy; 8 Department of Pharmacology, Dalhousie University, Halifax, NS, Canada

**Keywords:** 16S rRNA, bipolar disorder, gut microbiota, lithium, microbiota gut–brain axis, next generation sequencing, patient-centered approach

## Abstract

Lithium, the gold-standard treatment for bipolar disorder, exhibits highly heterogeneous clinical responses and no validated biological predictors of responsiveness are currently available. Emerging evidence suggests that the gut microbiota influences mood disorders and psychotropic drug response, raising the hypothesis that specific microbial signatures may modulate lithium responsiveness. In this study, we characterized taxonomic and functional gut microbiota profiles in 77 patients with bipolar disorder, of whom 40 were receiving lithium (20 responders, 20 non-responders) and 37 were treated with valproate as the main mood stabilizer (valproate, *n* = 31; lamotrigine, *n* = 6), with the aim of identifying potential microbial markers of clinical response. Microbiota composition was assessed through 16S rRNA sequencing targeting the V3-V4 region. Differential abundance was evaluated using Analysis of Composition of Microbiomes with Bias Correction (ANCOM-BC2), and the functional potential was inferred using the Phylogenetic Investigation of Communities by Reconstruction of Unobserved States 2 (PICRUSt2). Our finding showed that lithium treatment was associated with a selective gut microbiota reorganization, including reduced Actinobacteria (Actinomycetota) phylum, notably Coriobacteriia class, and enrichment in Firmicutes (Bacillota) taxa, including Selenomonadales, *Megamonas* and Clostridia taxa, alongside reductions in primary fermentative and biosynthetic pathways. This shift, characterized by a reduction of primary fermenters and enrichment of secondary fermenters and SCFA-producing taxa, suggests a more efficient fermentative ecosystem in lithium-treated patients. Responders showed enrichment in methanogenic taxa (*Methanobrevibacter*) and *Clostridiales vadinBB60* group when compared with patients treated with other mood stabilizers; however, these differences were not observed in the direct comparison between lithium responders and non-responders. While causal relationships cannot be inferred, these findings indicate treatment-associated microbial patterns and support further investigation into microbiota-directed adjunctive therapies.

## 1 Introduction

Lithium has represented for decades the reference pharmacological intervention for bipolar disorder (BD) and still remains the mainstay treatment, being able to significantly reduce the risk of relapse and suicide in affected individuals (Paribello et al., 2024). However, clinical response to long-term Li treatment is highly heterogeneous, its mechanism of action is only partly understood, and recent meta-analyses have shown that, despite numerous molecular and pharmacogenomic studies, no biomarkers have yet been universally validated to predict Li responsiveness (Paribello et al., 2024; Vecera et al., 2021).

In parallel, in recent years, research on the gut microbiota (GM) has highlighted its central role in modulating the gut-brain axis, influencing psychopathological phenotypes and the response to psychotropic treatments (Šimunović Filipčić et al., 2025). Review and experimental studies have reported that alterations of the GM are associated with mood disorders, including major depressive disorder and BD (Zhao J. et al., 2024), and that psychiatric medications, including Li, may influence the microbial profile (Bui et al., 2025; Minichino et al., 2024). Together, these findings open the possibility that the GM may harbor predictive biomarkers of pharmacological response or act as an active modulator of therapeutic efficacy.

Lithium exerts multifactorial pharmacological effects through the modulation of multiple targets, including GSK-3β (Glycogen Synthase Kinase-3 beta) and enzymes of the inositol pathway (Quiroz et al., 2004), through which indirectly affects a number of pathways and mechanism that lead to neurotrophic, neuroprotective and anti-inflammatory properties (Damri and Agam, 2024; de Sousa et al., 2011; Frey et al., 2006; Fukumoto et al., 2001; Ghanaatfar et al., 2023; Mehrafza et al., 2019; Sakrajda et al., 2025; Valvassori et al., 2015). Given these pleiotropic hallmarks it is reasonable to hypothesize that Li may also influence the brain-gut axis. Supporting this hypothesis, preclinical studies indicate that Li carbonate can mitigate intestinal mucosal inflammation by modulating the GM and activating regulatory T cells (Treg) through GPR43-dependent mechanisms, leading to an increase in Short-Chain Fatty Acids (SCFAs)-producing bacteria (Huang et al., 2022).

An *in vivo* experimental study conducted in a porcine model, evaluating different dietary sources of Li (Li carbonate and Li-enriched mushrooms) showed that therapeutic Li doses led to a significant increase in intestinal microbial diversity and richness, particularly in the colon, and to an enrichment of taxa considered beneficial for host health, such as *Lactobacillus*, Ruminococcaceae, *Enterorhabdus*, Muribaculaceae, and *Coprococcus* in pigs. Additional taxa, such as Prevotellaceae and Bacteroidales, were enriched in pigs receiving Li-enriched mushrooms, while Li carbonate increased Clostridia, *Ruminococcus*, *Burkholderia* and Bacteroidales at recommended dosages (de Souza Lopes et al., 2024).

Cussotto et al. (2019) similarly showed that Li, valproate and aripiprazole increased microbial diversity and richness in animal models without impairing intestinal barrier function, with enrichment in *Clostridium*, *Peptoclostridium*, *Intestinibacter*, and Christensenellaceae (Cussotto et al., 2019).

A recent systematic review on pharmacological treatments in BD reported that Li and antipsychotics were associated with significant variations in intestinal bacterial genera. While SCFA-producing species such as *Eubacterium biforme*, *E. rectale* and *Clostridium* Cluster IV, involved in maintaining the integrity of the intestinal barrier and in modulating the inflammatory response, were favored, opportunistic taxa such as *Clostridium perfringens*, *Klebsiella pneumoniae* and *Campylobacter hominis* increased in other contexts, suggesting possible therapy-associated dysbiosis (Bui et al., 2025). These mixed findings highlight the complex and potentially bidirectional effects of Li on the GM and reinforce the need for targeted studies in patients with BD.

Current evidence therefore supports the hypothesis that psychotropic treatments, including Li, may promote the growth of beneficial bacteria; however, findings remain conflicting and preliminary. These discrepancies likely reflect heterogeneity in study populations, stratification by dietary habits, treatment duration, metabolic comorbidities, concomitant medications and other covariates.

Furthermore, given the gastrointestinal and systemic side effects of Li (Volkmann et al., 2020) and its interactions with diet, medications and comorbidities, its influence on the GM may not be uniformly beneficial. Such side effects could either be independent of its microbiota-modifying properties or, conversely, could potentially serve as indicators for therapy monitoring (Ortega et al., 2023). It is plausible that Li promotes a favorable microbial balance in some patients, whereas in others, particularly in non-responders or patients with gastrointestinal side effects, the impact may be minimal or even unfavorable.

In this context, investigating whether the microbiota is differentially modulated in Li-responders and non-responders is essential, as such information may help identify microbial signatures predictive of therapeutic success.

Valproic acid (VPA), frequently used as a clinical comparator in clinical trials for mood stabilization, also exhibits diverse molecular mechanisms, including anticonvulsant and epigenetic actions related to histone deacetylases inhibition, and can affect metabolism and the microbiome (Poolchanuan et al., 2020). Consequently, VPA represents an appropriate comparison group to evaluate how two mood stabilizers with distinct mechanisms and safety profiles influence the GM.

From a clinical and translational perspective, comparing the GM profile of patients with BD treated with Li to that of patients treated with valproic acid as the main mood stabilizers, while stratifying responders and non-responders to Li, has three potential relevant outcomes: I) identifying microbial biomarkers associated with Li response to support personalized medicine; II) clarifying microbiota–metabolite–immune mechanisms influencing Li efficacy and tolerability; III) enabling future microbiota-targeted interventions, such as dietary strategies, probiotics, prebiotics and microbial therapeutics, to complement pharmacological treatment.

The existing literature on interactions between psychiatric pharmacology and the microbiome suggests a promising yet still emerging field, with a clear need for well-characterized cohort studies that rigorously control for confounders, such as drug exposure, treatment duration, disease history, metabolic variables, diet and antibiotic use.

To explore these interactions, we compared the GM profile of patients with BD treated with Li (*n* = 40) with a control group treated with other mood stabilizers (valproate, *n* = 31; lamotrigine, *n* = 6). We further stratified Li-treated patients into responders (*n* = 20) and non-responders (*n* = 20) to identify candidate microbial signatures associated with therapeutic responsiveness. This design allowed us to investigate whether specific microbial signatures differ in relation to both Li treatment and clinical response.

Note that the lack of an untreated control group is largely due to the difficulty in recruiting truly drug-naïve patients, as pharmacological treatment is often initiated before a definitive diagnostic assessment is completed. It would have allowed a more precise discrimination between microbiota alterations potentially related to bipolar disorder and those associated with pharmacological exposure.

## 2 Materials and methods

### 2.1 Study design and characteristics of the sample

Patients recruited by trained clinical psychopharmacologists and psychiatrists at the Unit of Clinical Pharmacology, University Hospital Agency of Cagliari, and at the Unit of Clinical Psychiatry of the University of Cagliari. The recruitment pipeline, diagnostic tools, and inclusion and exclusion criteria were agreed between the two centers and were as such harmonized. Patients had a diagnosis of BD type I or BD type II according to the criteria of the Diagnostic and Statistical Manual of Mental Disorders, 5th edition (APA) (Severus and Bauer, 2013). The exclusion criteria included the presence of other psychiatric or severe organic disorders, ongoing chemo/radiotherapy, severe psychopathology, gastrointestinal diseases, treatment with corticosteroids, proton pump inhibitors, antimicrobials, antibiotics, prebiotics and/or probiotic in the 3 months preceding sample collection, and assumption of substance abuse.

The study included 77 patients, of whom 40 were receiving Li at the time of recruitment (20 responders and 20 non- responders), while 37 were treated with other mood stabilizers (valproate, *n* = 31; lamotrigine, *n* = 6).

At recruitment, patients had been euthymic for at least 1 year and were stable on their current treatment. After enrollment, patients underwent multidimensional assessment, including clinical, anthropometric and lifestyle evaluation. At the same time, GM composition and predicted functional activity were assessed from fecal samples collected during the euthymic phase.

Patients receiving maintenance treatment with Li or other mood stabilizers were further stratified according to long-term treatment response. Response to treatment was evaluated using the Retrospective Criteria of Long-Term Treatment Response in Research Subjects with Bipolar Disorder (Alda scale) (Grof et al., 2002), which provides a validated quantitative measure of clinical improvement during mood stabilizer treatment, with total scores ranging from 0 to 10 after adjustment for potential confounding factors. Briefly, this scale quantifies the degree of improvement in the course of treatment (A criterion or A score) expressed as a composite measure of change in frequency and severity of mood symptoms. The A score is weighed against 5 factors (B criteria) which allow one to determine if the observed improvement is a result of the treatment rather than a spontaneous improvement or an effect of additional medication. Specifically, the B criteria consider: the number of episodes before/off the treatment (B1), the frequency of episodes before/off the treatment (B2), the duration of the treatment (B3), the compliance during period(s) of stability (B4) and the use of additional medication during the period of stability (B5). The total score (TS) is obtained by subtracting the B score from the A score.

Based on the results of the inter-rater agreement (Manchia et al., 2013), a total score ≥7 was considered indicative of a favorable response (Grof et al., 2002; Manchia et al., 2013). More specifically, the study by Manchia et al. (2013), which evaluated the inter-rater agreement [Kappa (κ)] of twenty-nine ConLiGen sites who took part in a two-stage case-vignette rating procedure, showed that the highest K was obtained for the TS cut off point of 7. This confirmed what was previously shown by Grof et al. (2002), further supporting the use of this cut-off for binary definition of full response to lithium and to other mood stabilizers. This same value has been used and validated in many other studies across the years (Camarena et al., 2025; Miola et al., 2026). Considering the small number of responders in the group of non-Li treated patients (responders to valproic acid, *n* = 3; responders to lamotrigine, *n* = 1), responders and non-responders to Li were compared with overall sample of patients treated with other mood stabilizers for both clinical variables and gut microbial profile.

### 2.2 Clinical, anthropometric, and lifestyle evaluation

All patients underwent clinical assessment, including age, sex, age at disease onset, illness duration in years, bipolar sub-diagnosis, treatment response status, use of concomitant mood stabilizers and number of depressive and manic episodes. Anthropometric evaluation, including measurement of Body Mass Index (BMI), was performed following current standards (Lohman et al., 1992), as previously described (Pisanu et al., 2020).

Sex was recorded as a biological variable (male/female) based on sex assigned at birth as reported in the medical records. In this study, the term sex refers to biological attributes (chromosomal, hormonal and anatomical characteristics), while gender identity was not assessed.

Age was categorized in two different ways. First, participants were divided into three groups: early adulthood (25–45 years), midlife (46–60 years) and late life ( >60 years). Second, age was dichotomized into two groups: adults (25–60 years) and late life ( >60 years).

Among lifestyle factors, physical activity, smoking status, adherence to the Mediterranean diet (assessed through its validated score) were evaluated. Comorbidities were additionally recorded. Nutritional assessment was based on administration of the standardized and validated Mediterranean Diet Score, (MDS, range 0–55) questionnaire (Martínez-González et al., 2012).

### 2.3 Gut microbiota analysis

#### 2.3.1 Sample collection

A stool sample from each patient was self-collected during the euthymic phase and delivered to the laboratory within 3 h. Fresh samples were aliquoted and stored at -80°C until further processing.

#### 2.3.2 Genomic DNA extraction, bacterial DNA quantification, and 16S libraries preparation and sequencing

Genomic DNA extraction from stool samples and bacterial DNA quantification were performed as previously described (Santoru et al., 2018). The library preparation protocol has been detailed elsewhere (Palmas et al., 2021). Barcoded 16S amplicon libraries targeting the V3-V4 hypervariable region of the bacterial 16S rRNA gene were generated using specific primers and the Nextera XT index kit (Illumina, Inc., San Diego, CA, United States). Library size and quality were verified using the D1000 reagents kit (Agilent Technologies, Santa Clara, CA, United States) on the Tapestation 4200 system (Agilent Technologies, Santa Clara, CA, United States). Quantification, normalization, pooling of 16S libraries and the PhiX control preparation were performed as previously described (Deledda et al., 2022; Palmas et al., 2025). The combined 16S library and PhiX control were denatured and sequenced on the MiSeq Illumina platform using a v3 paired-end run (2 × 300 cycles) and the MiSeq v3 Reagent Kit (Illumina).

### 2.4 Bioinformatic and statistical analysis

Participant data were summarized as mean ± standard deviation (SD) for continuous variables and n (%) for categorical variables. Between-group comparisons were performed using Welch’s *t*-test or ANOVA for normally distributed continuous variables, Mann–Whitney U or Kruskal-Wallis tests for non-normally distributed variables, and Chi-square or Fisher’s exact tests for categorical variables. *Post-hoc* pairwise comparisons with Benjamini-Hochberg correction were applied when global differences were significant. Effect sizes were calculated for significant comparisons. Statistical significance was set at *p* < 0.05.

Sequences were processed using QIIME2 (Bolyen et al., 2019). Quality control was performed using FastQC (Wingett and Andrews, 2018) and MultiQC (Ewels et al., 2016). Primers were removed using cutadapt (q2-cutadapt QIIME 2 plugin) (Martin, 2011). Using DADA2 (q2-dada2 QIIME 2 plugin), reads were trimmed and filtered by quality (Callahan et al., 2016). High-quality reads were denoised and merged to produce amplicon sequence variants (ASVs). ASVs were aligned using MAFFT (Katoh and Standley, 2013), and a phylogenetic tree was constructed using FastTree2 (Price et al., 2010) within the q2-phylogeny plugin of QIIME2. The p-sampling-depth parameter was set to 18,000, below the minimum read count across samples, ensuring normalization and comparability of data. Taxonomic assignment of ASVs was performed using a Naive-Bayes classifier through the q2-feature-classifier plugin of QIIME2, trained on the specific primer pairs used in this study and on the SILVA (v132) database (Quast et al., 2013). We are aware that some of the taxon names that we use have changed [for example, Actinobacteria is now named Actinomycetota; Euryarchaeota is now named Methanobacteriota (Göker et al., 2025)]. However, we chose to report the old names because they are those given in reference to the database that we used (Quast et al., 2013), thus ensuring consistency and reproducibility.

Alpha and beta diversity metrics were calculated using the q2-diversity plugin. Statistical differences in alpha diversity were assessed using the linear regression model for normally distributed metrics (Observed ASVs, Chao1, and Faith’s Phylogenetic Diversity), a beta regression model for metrics ranging between 0 and 1 (Evenness and Simpson), and rank-based linear model for non-normal distribution (Shannon), adjusting for relevant clinical covariates. The beta diversity was visualized through Principal Coordinates Analysis (PCoA). Dotted ellipses indicated 95% confidence intervals for a multivariate t-distribution, computed with the stat ellipse function from ggplot2 (R package) (Valero-Mora, 2010). Significance differences in beta diversity distances were tested using PERMANOVA test, with the adonis2 function from the vegan package in R, with *p* ≤ 0.05 considered statistically significant.

Differential abundance analysis was performed using ANCOM-BC2 (Analysis of Composition of Microbiomes with Bias Correction v.2.9.1) in R (Lin and Peddada, 2024). The analysis was controlled for potential confounders, including age, years of illness, years of treatment, use of additional mood stabilizers, antipsychotic treatment at recruitment, number of manic episodes, total number of episodes, physical activity, MedDiet score and hypertension.

Predicted functional metagenomic pathways were inferred using the Phylogenetic Investigation of Communities by Reconstruction of Unobserved States 2 (PICRUSt2 v.2.5.2) (Douglas et al., 2020) and classified according to the Metabolic Pathway (MetaCyc) database (Caspi et al., 2020). Statistical comparisons of pathway abundances were performed using the ANOVA-Like Differential Expression (ALDEx2) (Fernandes et al., 2013; Fernandes et al., 2014), using the Wilcoxon rank-sum test to compare two groups (patients treated with Li and patients treated with other mood stabilizer) and the generalized linear model (glm) function to compare three groups (Li-responders, Li non-responders, and patients treated with other mood stabilizer). Benjamini-Hochberg correction was applied for multiple testing. All microbiome analyses (statistics and plotting) were performed in R, and all plots were generated using the *ggplot2* package (Valero-Mora, 2010).

## 3 Results

### 3.1 Anthropometric, metabolic, lifestyle, and health status evaluation

The study included 77 patients: 40 (14 men and 26 women) were treated with Li and 37 (15 men and 22 women) were treated with valproic acid as main mood stabilizer (see Table 1). The two groups did not differ for sex, BMI, smoking status, adherence to the Mediterranean diet, age at illness onset, subtype diagnosis, treatment response, use of other mood stabilizers, number of depressive episodes, and presence of diabetes.

**TABLE 1:** Comparison of demographic, clinical, anthropometric, lifestyle, and comorbidity characteristics among patients treated with Li and those treated with other mood stabilizers.

Variable	Li-treated (*n* = 40)	Other mood stabilizers (*n* = 37)	Statistical test used	Test (*t*, *W*, or χ^2^)	*P*-value
Sex (*n*; %)			Chi-square	χ^2^ = 0.10751	0.743
Man	14; 35.00	15; 40.54			
Woman	26; 65.00	22; 59.46			
Age (M ± SD)	57.45 ± 12.53	50.30 ± 10.06	Welch’s *t*-test	*t* = 2.7712	0.007**
Age (*n*; %)			Chi-square	χ^2^ = 10.615	0.005**
Early adulthood	9; 22.50	10; 27.03			
Midlife	11; 27.50	21; 56.76			
Late life	20; 50.00	6; 16.22			
Age (*n*; %)			Chi-square	χ^2^ = 8.3566	0.004**
Adult	20; 50.00	31; 83.78			
Late life	20; 50.00	6; 16.22			
Age at onset of the disease (M ± SD)	25.67 ± 8.63	28.62 ± 9.19	Mann–Whitney U	W = 601.5	0.159
Illness in years (M ± SD)	31.77 ± 12.70	21.67 ± 11.53	Welch’s *t*-test	*t* = 3.6578	0.0005**
Sub-diagnosis (*n*; %)			Chi-square		0.867
BDI	24; 60.00	25; 67.57			
BDII	13; 32.50	11; 29.73			
Treatment responders (*n*; %)					
Yes	20; 50.00	3; 8.11			
No	20; 50.00	28; 75.68			
Use of additional mood stabilizers (*n*; %)			Chi-square	χ^2^ = 1.4176	0.234
Yes	13; 32.50	6; 16.22			
No	27; 67.50	28; 75.68			
Number of depressive episodes (M ± SD)	7.76 ± 7.11	10.59 ± 10.00	Mann–Whitney U	W = 509	0.234
Number of manic episodes (M ± SD)	7.34 ± 7.16	3.68 ± 4.85	Mann–Whitney U	W = 891	0.006**
Anthropometric data					
BMI (M ± SD)	25.93 ± 4.34	27.68 ± 6.06	Mann–Whitney U	W = 569	0.316
BMI (*n*; %)			Chi-square	χ^2^ = 1.3815	0.501
Normal weight	20; 50.00	12; 32.43			
Overweight	12; 30.00	13; 35.13			
Obesity	8; 20.00	8; 21.62			
Lifestyle factors					
Physical activity (*n*; %)			Chi-square	χ^2^ = 3.9231	0.048*
No	20; 50.00	26; 70.27			
Yes	19; 47.50	8; 21.62			
Smoking status Yes (*n*; %)			Chi-square	χ^2^ = 1.3271	0.249
No	16; 40.00	20; 54.05			
Yes	23; 57.50	15; 40.54			
MedDietAdherence (*n*; %)			Fisher’s exact	-	0.770
High	5; 12.50	5; 13.51			
Moderate	30; 75.00	25; 67.57			
Low	5; 12.50	7; 18.92			
MedDietScore (M ± SD)	7.65 ± 1.82	7.32 ± 2.34	Mann–Whitney U	W = 810	0.473
Comorbidities					
Diabetes (*n*; %)			Fisher’s exact	-	1.000
No	38; 95.00	36; 97.30			
Yes	2; 5.00	1; 2.70			
Hypertension (*n*; %)			Fisher’s exact	-	0.015*
No	31; 77.50	36; 97.30			
Yes	9; 22.50	1; 2.70			

Data are expressed as mean (M) ± Standard Deviation (SD) for continuous variables and as number (percentage) for categorical variables. Between-group comparisons were performed using the Welch’s *t*-test for normally distributed continuous variables, the Mann–Whitney U test for non-normally distributed variables, and the Chi-square or Fisher’s exact test for categorical variables. Significant results (*p* < 0.05) are highlighted in bold. *: statistically significant with adjusted *p*-value < 0.05; **: statistically significant with adjusted *p*-value < 0.01. M, Mean; SD, Standard Deviation; Li, Lithium; BMI, Body Mass Index; BD, Bipolar Disorder; BDI, Bipolar Disorder I; BDII, Bipolar Disorder II; MedDiet, Mediterranean Diet. t, Welch’s *t*-test statistic; W, Mann–Whitney (Wilcoxon rank-sum) test statistic; χ^2^ = chi-square test.

Significant differences were found with respect to age, both when considered as a continuous variable and when stratifying the study population into age groups. Most participants were adults, with a higher representation of late life among patients treated with Li (50%) and midlife among those treated with other mood stabilizers (56.76%). Using the dichotomized age classification, half of the Li-treated participants were adults (25–60 years) and half belonged to the late-life group ( >60 years), whereas 83.8% of participants treated with other mood stabilizers were adults and 16.2% were classified as late-life group. Additional significant differences between the two cohorts regarding illness duration (in years), number of manic episodes, level of physical activity and presence of hypertension were observed.

When stratifying the study population of patients treated with Li into groups according to their response to therapy (see Table 2), among Li-responders six patients (30%) were men and 13 (65%) were women, while among non-responders, seven (35%) were men and 13 (65%) were women. Significant age differences were observed between groups, mainly driven by the comparison between Li-responders and patients treated with other mood stabilizers, with a mean age of 60.25 years in Li-responders, compared to 54.65 years in Li non-responders and 50.30 years in the group treated with other mood stabilizers.

**TABLE 2:** Comparison of demographic, clinical, anthropometric, lifestyle, and comorbidity characteristics among Li-responders (Li R), Li non-responders (Li NR), and patients treated with other mood stabilizers.

Variable	Li R (*n* = 20)	Li NR (*n* = 20)	Other mood stabilizers (*n* = 37)	Statistical test; correction method	F or χ^2^ statistic	Effect size method	Global *p*-value	*Post-hoc* pairwise comparisons	Effect size
Sex (*n*; %)				Pearson’s Chi-squared; BH	0.47286	Cramer’s V with MC	0.789	-	
Man	7; 35.00	7; 35.00	15; 40.54						
Woman	13; 65.00	13; 65.00	22; 59.46						
Age (M ± SD)	60.25 ± 11.85	54.65 ± 12.87	50.30 ± 10.06	ANOVA; Tukey HSD	5.075	Cohen’s d	**0.009**	Li R vs. Li NR: 0.267; Li R vs. Other: 0.006**; Li NR vs. Other: 0.353	Li R vs. Li NR: 0.453; Li R vs. Other: 0.929; Li NR vs. Other: 0.392
Age (*n*; %)				Fisher’s exact; BH		Cramer’s V with Monte Carlo	**0.013**	Li R vs. Li NR: 0.356; Li R vs. Other: 0.016*; Li NR vs. Other: 0.124	Li R vs. Li NR: 0.217; Li R vs. Other: 0.451; Li NR vs. Other: 0.296
Early adulthood	3; 15.00	6; 30.00	10; 27.03						
Midlife	5; 25.00	6; 30.00	21; 56.76						
Late life	12; 60.00	8; 40.00	6; 16.22						
Age (*n*; %)				Pearson’s Chi-squared; BH	11.598	Cramer’s V with Monte Carlo	**0.003**	Li R vs. Li NR: 0.206; Li R vs. Other: 0.002**; Li NR vs. Other: 0.070	Li R vs. Li NR: 0.200; Li R vs. Other: 0.450; Li NR vs. Other: 0.264
Adult	8; 40.00	12; 60.00	31; 83.78						
Late life	12; 60.00	8; 40.00	6; 16.22						
Age at onset of the disease (M ± SD)	24.25 ± 8.22	27.10 ± 9.00	28.62 ± 9.19	Kruskal-Wallis; Wilcoxon BH	3.3705	Rank Biserial	0.185	-	
Illness in years (M ± SD)	36.00 ± 12.86	27.55 ± 11.31	21.67 ± 11.53	ANOVA; Tukey HSD	9.553	Cohen’s d	**0.0002**	Li R vs. Li NR: 0.068; Li R vs. Other: 0.0001**; Li NR vs. Other: 0.018	Li R vs. Li NR: 0.698; Li R vs. Other: 1.193; Li NR vs. Other: 0.513
Years of treatment	19.75 ± 10.19	18.53 ± 10.46	11.41 ± 6.59	Kruskal-Wallis; Wilcoxon BH	9.5107	Rank Biserial	**0.009**	Li R vs. Li NR: 0.746; Li R vs. Other: 0.010*; Li NR vs. Other: 0.037*	Li R vs. Li NR: 0.060 95% CI: [-0.29, 0.41]; Li R vs. Other: 0.530 95% CI: [0.23, 0.74]; Li NR vs. Other: 0.410 95% CI: [0.08, 0.66]
Sub-diagnosis (*n*; %)				Pearson’s chi-squared; BH	0.64024	Cramer’s V with Monte Carlo	0.726	-	
BDI	12; 60.00	12; 60.00	25; 67.57						
BDII	8; 40.00	5; 25.00	11; 29.73						
Use of additional mood stabilizers (*n*; %)				Fisher’s exact; BH	12.736	Cramer’s V with Monte Carlo	**0.004**	Li R vs. Li NR: 0.010*; Li R vs. Other: 0.695; Li NR vs. Other: 0.010*	Li R vs. Li NR: 0.480; Li R vs. Other: 0.104; Li NR vs. Other: 0.388
Yes	2; 10.00	11; 55.00	6; 16.22						
No	18; 90.00	9; 45.00	28; 75.68						
Antipsychotic treatment at recruitment				Pearson’s Chi-squared; BH	22.731	Cramer’s V with Monte Carlo	**8,00E-06**	Li R vs. Li NR: 4.98E-05**; Li R vs. Other: 0.001**; Li NR vs. Other: 0.174	Li R vs. Li NR: 0.704; Li R vs. Other: 0.489; Li NR vs. Other: 0.225
Yes	4; 20.00	18; 90.00	24; 67.86						
No	16; 80.00	2; 10.00	10; 27.03						
Antidepressants treatment at recruitment				Fisher’s Exact; BH	Cramer’s V with Monte Carlo	0.683	–	–
Yes	3; 15.00	5; 25.00	9; 24.32					
No	17; 85.00	15; 75.00	25; 67.57					
Number of depressive episodes (M ± SD)	9.38 ± 8.13	6.24 ± 5.83	10.59 ± 10.00	Kruskal-Wallis; Wilcoxon BH	2.7234	Rank Biserial	0.256	–	–
Number of manic episodes (M ± SD)	8.71 ± 9.32	6.06 ± 4.15	3.68 ± 4.85	Kruskal-Wallis; Wilcoxon BH	7.9569	Rank Biserial	**0.019**	Li R vs. Li NR: 0.921; Li R vs. Other: 0.164; Li NR vs. Other: 0.014*	Li R vs. Li NR: -0.020, -0.02 95% CI: [-0.39, 0.35]; Li R vs. Other: 0.270 95% CI: [-0.05, 0.55]; Li NR vs. Other: 0.470 95% CI: [0.18, 0.69]
Total number of episodes (M ± SD)	39.15 ± 16.86	37.85 ± 14.51	15.81 ± 12.76	Kruskal-Wallis; Wilcoxon BH	35.416	Rank Biserial	**2,00E-08**	Li R vs. Li NR: 0.989; Li R vs. Other: 2.00E-6**; Li NR vs. Other: 2.00E-6**	Li R vs. Li NR: -0.005, -0.005 95% CI: [-0.35, 0.34]; Li R vs. Other: 0.790 95% CI: [0.65, 0.89]; Li NR vs. Other: 0.780 95% CI: [0.63, 0.88]
Anthropometric data									
BMI (M ± SD)	26.07 ± 4.06	25.79 ± 4.71	27.68 ± 6.06	Kruskal-Wallis; Wilcoxon BH	1.0241	Rank Biserial	0.599	–	–
BMI (*n*; %)				Fisher’s exact; BH	–	Cramer’s V with Monte Carlo	0.922	–	–
Normal weight	10; 50.00	9; 45.00	12; 32.43						
Overweight	6; 30.00	5; 25.00	13; 35.13						
Obesity	4; 20.00	4; 20.00	8; 21.62						
Lifestyle factors									
Physical activity (*n*; %)				Pearson’s Chi-squared; BH	14.854	Cramer’s V with Monte Carlo	**0.001**	Li R vs. Li NR: 0.003**; Li R vs. Other: 0.001**; Li NR vs. Other: 0.903	Li R vs. Li NR: 0.487; Li R vs. Li Other: 0.488; Li NR vs. Other: 0.017
No	5; 25.00	15; 75.00	26; 70.27						
Yes	14; 70.00	5; 25.00	8; 21.62						
Smoking status Yes (*n*; %)				Pearson’s Chi-squared; BH	5.1271	Cramer’s V with Monte Carlo	0.077	–	–
No	5; 25.00	11; 55.00	20; 54.05						
Yes	14; 70.00	9; 45.00	15; 40.54						
MedDietAdherence (*n*; %)				Fisher’s exact; BH	–	Cramer’s V with Monte Carlo	0.928	–	–
High	3; 15.00	2; 10.00	5; 13.51						
Moderate	15; 75.00	15; 75.00	25; 67.57						
Low	2; 10.00	3; 15.00	7; 18.92						
MedDietScore (M ± SD)	8.35 ± 1.60	6.95 ± 1.79	7.32 ± 2.34	Kruskal-Wallis; Wilcoxon BH	6.4217	Rank Biserial	**0.040**	Li R vs. Li NR: 0.025*; Li R vs. Other: 0.102; Li NR vs. Other: 0.525	Li R vs. Li NR: 0.480 95% CI: [0.16, 0.71]; Li R vs. Other: 0.290 95% CI: [-0.02, 0.55]; Li NR vs. Other: -0.100 95% CI: [-0.40, 0.21]
Comorbidities									
Diabetes (*n*; %)				Fisher’s exact	–	Cramer’s V with Monte Carlo	1.000	–	–
No	19; 95.00	19; 95.00	36; 97.30						
Yes	1; 5.00	1; 5.00	1; 2.70						
Hypertension (*n*; %)				Fisher’s exact; BH	–	Cramer’s V with Monte Carlo	**0.008**	Li R vs. Li NR: 0.451; Li R vs. Other: 0.017*; Li NR vs. Other: 0.178	Li R vs. Li NR: 0.180; Li R vs. Li Other: 0.397; Li NR vs. Other: 0.230
No	14; 70.00	17; 85.00	36; 97.30						
Yes	6; 30.00	3; 15.00	1; 2.70						

Clinical, demographic, anthropometric, lifestyle, and comorbidity characteristics of participants by treatment group (Li-Responders, Li Non-Responders and Other mood stabilizers). Continuous variables are presented as mean ± SD and categorical variables as n (%). Global *p*-values indicate the overall differences among groups. *Post-hoc* pairwise comparisons are shown only for variables with statistically significant global *p*-values. Effect sizes are reported for pairwise comparisons. Significant results (*p* < 0.05) are highlighted in bold, as *p < 0.05, **p < 0.01. Categorical variables were analyzed with Pearson’s Chi-squared or Fisher’s Exact tests (BH correction for multiple comparisons). Continuous variables were tested for normality (Shapiro-Wilk); normally distributed variables were analyzed using ANOVA with Tukey HSD *post-hoc* tests, non-normal variables with Kruskal-Wallis and Wilcoxon *post-hoc* tests (BH correction). Effect sizes are reported for significant comparisons: Cohen’s d for ANOVA, rank biserial for Wilcoxon, and Cramer’s V for categorical variables. M, Mean; SD, Standard Deviation.

When stratifying by age category, significant differences between Li-responders and patients treated with other mood stabilizers were also detected. The majority of Li-responders were classified as late life group (60%), while Li non-responders were more evenly distributed across early adulthood (30%), midlife (30%), and late life (40%). In contrast, patients treated with other mood stabilizers were predominantly in midlife (56.8%), with a smaller proportion classified as late life group (16.2%). These findings suggest that older age was more frequent among Li-responders, whereas younger and middle-aged adults were more represented in the group treated with alternative stabilizers.

Similarly, significant between-group differences were observed for illness duration, treatment duration, use of additional mood stabilizers, use of antipsychotics at recruitment, total number of mood episodes, total number of manic episodes, engagement in physical activity, Mediterranean diet score and prevalence of hypertension. A significantly higher proportion of Li non-responders (55%) used additional mood stabilizers, compared with Li-responders (10%) and patients treated with valproic acid as the main mood stabilizer (16.2%). Likewise, 90% of Li non-responders were taking antipsychotic medications, a proportion significantly higher than in Li-responders (20%), whereas Li-responders showed significantly lower use of antipsychotics compared with patients treated with other mood stabilizers (67.86%).

Conversely, a lower proportion of Li-responders (25%) reported engaging in physical activity compared with both non-responders (75%) and patients treated with other stabilizers (70.27%). However, adherence to the Mediterranean diet was significantly higher among Li-responders than among non-responders. Responders and non-responders also exhibited a significantly longer duration of treatment (in years) compared with patients treated with other mood stabilizers, as well as a higher number of total mood episodes. Furthermore, a greater proportion of Li non-responders were concurrently taking additional mood stabilizers compared with responders and with patients treated with valproic acid as the main mood stabilizer.

### 3.2 Gut microbiota analysis

Samples were normalized to 18,000 reads (the lowest number of reads per sample). After processing the reads through DADA2, approximately 75% passed the QC filter and ∼60% on average remained in the last step (passed QC, non-chimeric reads) (see Supplementary Figures 3, 4).

#### 3.2.1 Alpha and beta diversity analysis

**FIGURE 1:**
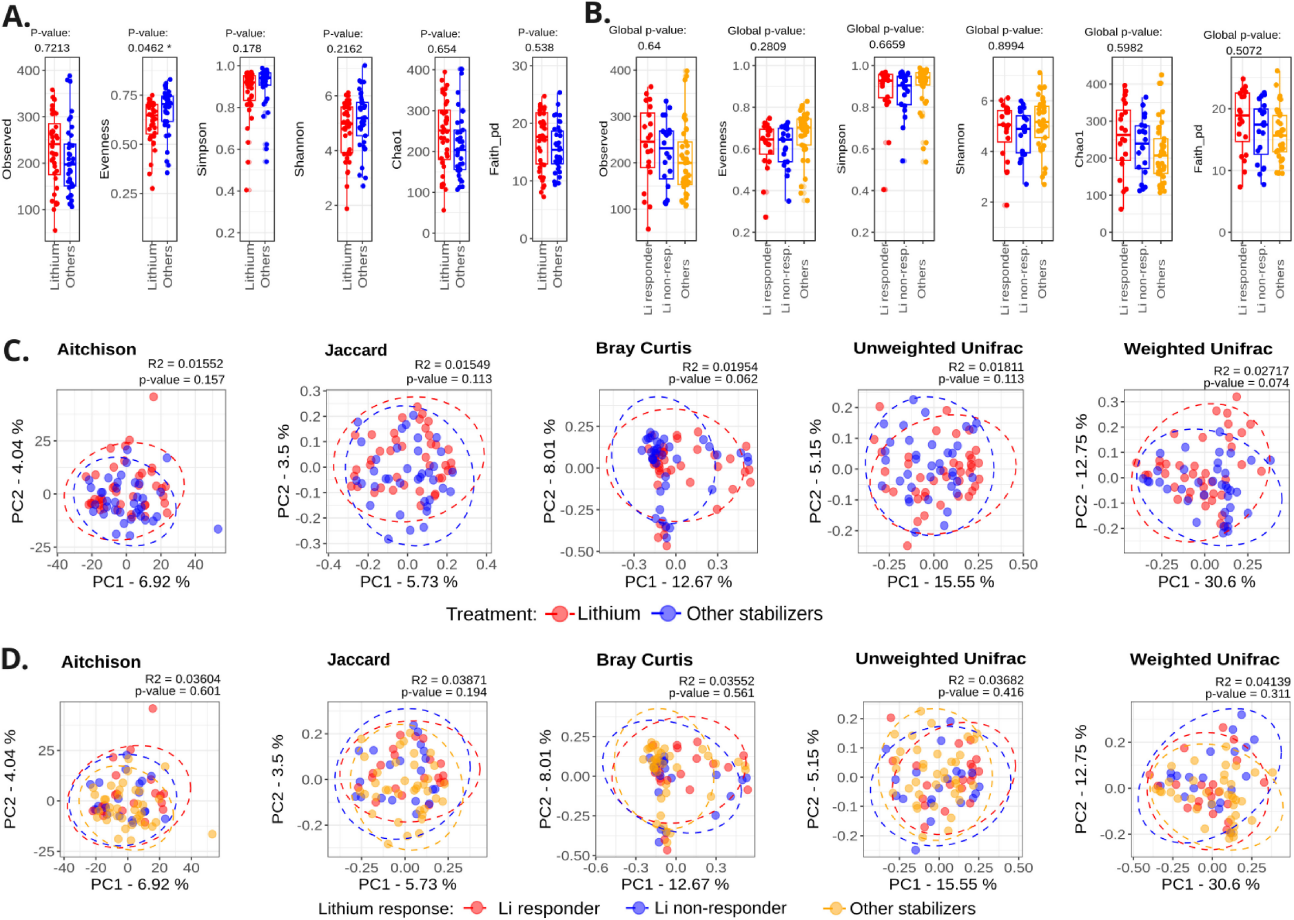
Alpha- and beta-diversity analysis between patients with BD treated with Li and patients treated with other mood stabilizers and according to the response to Li. **(A)** shows the alpha diversity analysis on the following metrics: Observed ASVs, Evenness (Pielou’s J), Simpson, Shannon, Chao1, and Faith’s Phylogenetic Diversity indices. The boxes indicate median values, and each dot corresponds to the alpha-diversity value of a single sample. Red dots represent samples from patients treated with Li, whereas blue dots represent samples from patients treated with other mood stabilizers. Statistical significance between groups was assessed using the linear regression model for metrics with normal distribution (Observed ASVs, Chao1, and Faith’s Phylogenetic Diversity), beta regression model for metrics between 0 and 1 (Evenness and Simpson), and rank-based linear model for non-normal distribution (Shannon), with relevant clinical covariates (age in years, years of illness, number of manic episodes, physical activity, and hypertension). Significance was set at *p* ≤ 0.05. **(C)** shows the Principal Coordinates Analysis (PCoA) based on Aitchison, Jaccard, Bray-Curtis, unweighted UniFrac, and weighted UniFrac distance matrices. Red and blue dots indicate samples from Li-treated patients and patients treated with other mood stabilizers, respectively. Dotted ellipses represent 95% confidence intervals for a multivariate t-distribution using the stat_ellipse function from the ggplot2 R package. Between-group differences in beta diversity were assessed using PERMANOVA, with relevant clinical covariates (age in years, years of illness, number of manic episodes, physical activity, and hypertension). A *p*-value ≤ 0.05 was considered statistically significant. ASV, Amplicon Sequence Variant; BD, Bipolar Disorder; FDR, False Discovery Rate. **(B)** The alpha diversity of GM in Li-responders (red), Li non-responders (blue), and patients treated with other mood stabilizers (orange) on the following diversity metrics: Observed ASVs, Pielou’s Evenness, Simpson, Shannon, Chao1, and Faith’s Phylogenetic Diversity. These metrics were compared between groups using linear regression model for metrics with normal distribution (Observed ASVs, Shannon, Chao1, and Faith’s Phylogenetic Diversity), and beta regression model for metrics between 0 and 1 (Evenness and Simpson), with relevant clinical covariates (age in years, years of illness, years of treatment, use of additional mood stabilizers, antipsychotic treatment at recruitment, number of manic episodes, total number of episodes, physical activity, MedDiet score, and hypertension). **(D)** Beta diversity distances visualized by Principal Coordinates Analysis (PCoA) based on Aitchison, Jaccard, Bray-Curtis, unweighted UniFrac, and weighted UniFrac distances in Li-responders (red), Li non-responders (blue), and patients treated with other mood stabilizers (orange). Ellipses indicate 95% confidence intervals. Global significance was assessed using PERMANOVA with relevant clinical covariates (age in years, years of illness, years of treatment, use of additional mood stabilizers, antipsychotic treatment at recruitment, number of manic episodes, total number of episodes, physical activity, MedDiet score, and hypertension).

Alpha diversity analysis did not reveal significant differences in species richness or overall diversity between patients treated with Li and those receiving other mood stabilizers. No statistically significant differences were observed for Observed ASVs (*p* = 0.7213), Shannon (*p* = 0.2162), Faith’s Phylogenetic Diversity (*p* = 0.538), Chao1 (*p* = 0.6540) and Simpson indices (*p* = 0.178). However, Pielou’s evenness was significantly lower in the Li-treated group compared to patients receiving other mood stabilizers (*p* = 0.0462), suggesting a less uniform distribution of microbial taxa in these individuals (see Figure 1A). Means, SD, F statistic, R-squared, and *p*-values of each analysis can be found in the Supplementary Table 1.

To visualize the beta diversity among the same groups, we obtained the PCoA using the following distances: Aitchison, Jaccard, Bray-Curtis dissimilarity, unweighted UniFrac, and weighted UniFrac metric (Figure 1C).

The first two axes of the PCoA explained approximately 20% of the variance for the unweighted UniFrac distance, while the variance explained for Bray-Curtis and weighted UniFrac distances was approximately 20 and 42%, respectively.

To assess whether the microbial community composition significantly differed between treatment groups, a PERMANOVA test was performed (see Supplementary Table 3). No significant differences were detected for Aitchison (*p* = 0.157), Jaccard (*p* = 0.113), Bray-Curtis (*p* = 0.062), unweighted UniFrac (*p* = 0.113) or weighted UniFrac (*p* = 0.074) distances.

We investigated the alpha and beta diversity of the GM across three clinical groups: patients who responded to Li treatment (Li-responders), patients who did not respond to Li (Li non-responders), and patients treated with other mood stabilizers (Other mood stabilizers). No significant differences were observed among groups for Observed ASVs (*p* = 0.6400), Pielou’s evenness (*p* = 0.2809), Shannon (*p* = 0.8994), Faith’s Phylogenetic Diversity (*p* = 0.5072), Chao1 (*p* = 0.5982) and Simpson indices (*p* = 0.6659) (see Figure 1B and Supplementary Table 2).

PCoA plots based on multiple distance metrics (Aitchison, Jaccard, Bray–Curtis, Unweighted UniFrac, and Weighted UniFrac) revealed an overall overlap in the microbial composition between groups. No significant differences were observed for Aitchison (*p* = 0.601), Jaccard (*p* = 0.194), Bray-Curtis (*p* = 0.561), unweighted UniFrac (*p* = 0.416) or weighted UniFrac (*p* = 0.311) distances (see Figure 1D and Supplementary Table 4).

#### 3.2.2 Compositional analysis of intestinal microbiota

**FIGURE 2:**
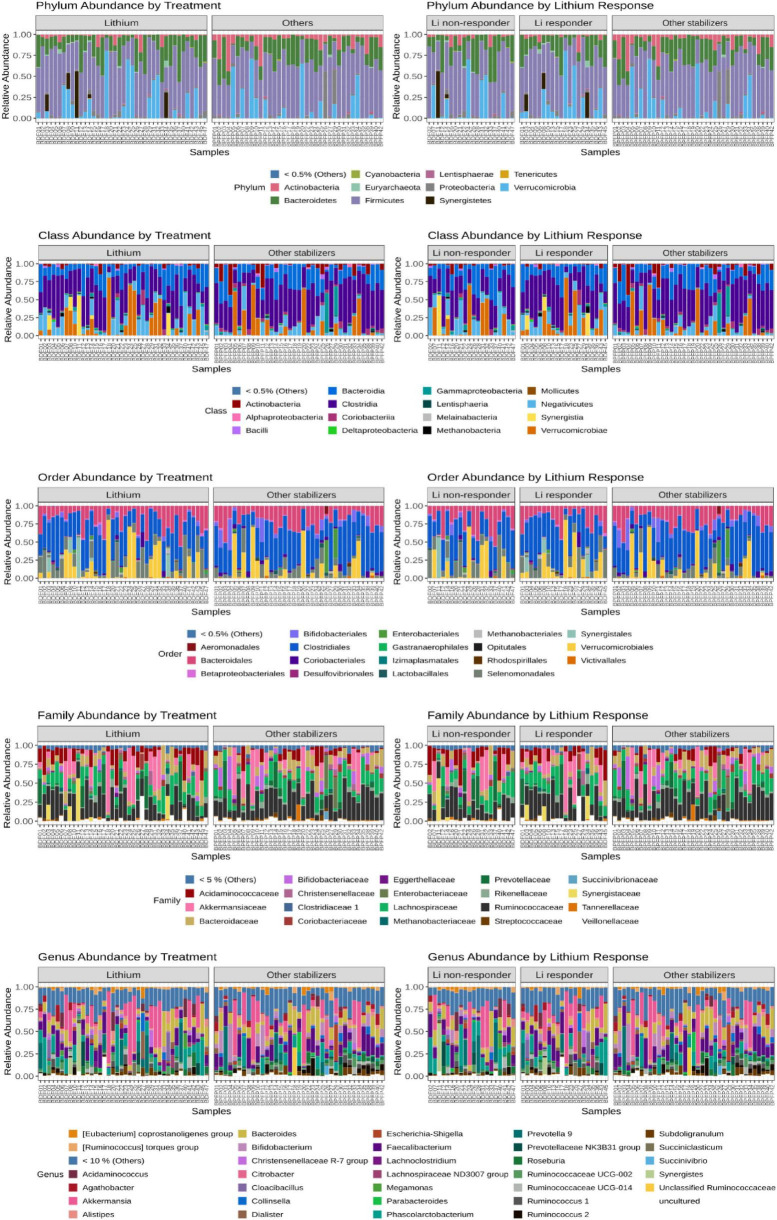
Taxonomic composition of gut microbiota in patients treated with Li versus other mood stabilizers. The stacked bar plots represent the relative abundance of microbiota at different taxonomic levels (phylum, class, order, family, genus) in different patient groups: lithium-treated (Li-treated), lithium responders (Li-responder), lithium non-responders (Li non-responder), and patients treated with valproic acid as main mood stabilizers (Other stabilizers). Each bar indicates the proportional distribution of different taxa within the microbiota, with colors corresponding to specific phyla, classes, orders, families and genera. Taxonomic annotations follow SILVA v132 nomenclature; updated ICNP-compliant phylum names are provided as follows: Actinobacteria (Actinomycetota), Firmicutes (Bacillota), Cyanobacteria (Cyanobacteriota), Lentisphaerae (Lentisphaerota), Proteobacteria (Pseudomonadota), Tenericutes (Mycoplasmatota), Verrucomicrobia (Verrucomicrobiota), Synergistetes (Synergistota), and Euryarchaeota (Methanobacteriota).

Taxonomic profiling of GM is shown in Figure 2. At the phylum level, both Li-treated patients and those on other mood stabilizers exhibited a predominance of Firmicutes (Bacillota) and Bacteroidota, with substantial interindividual variability. Interestingly, Actinobacteria (Actinomycetota) appeared to be more abundant in patients treated with other mood stabilizers compared to those treated with Li.

Further stratification of Li-treated patients into responders and non-responders revealed additional differences in microbiota composition. At the class and order levels, taxa such as Clostridia and Bacteroidia were dominant across groups.

At the family and genus levels, clearer divergences were observed. Families like Ruminococcaceae and Lachnospiraceae were abundant in all groups, though their proportions differed across subgroups.

**FIGURE 3:**
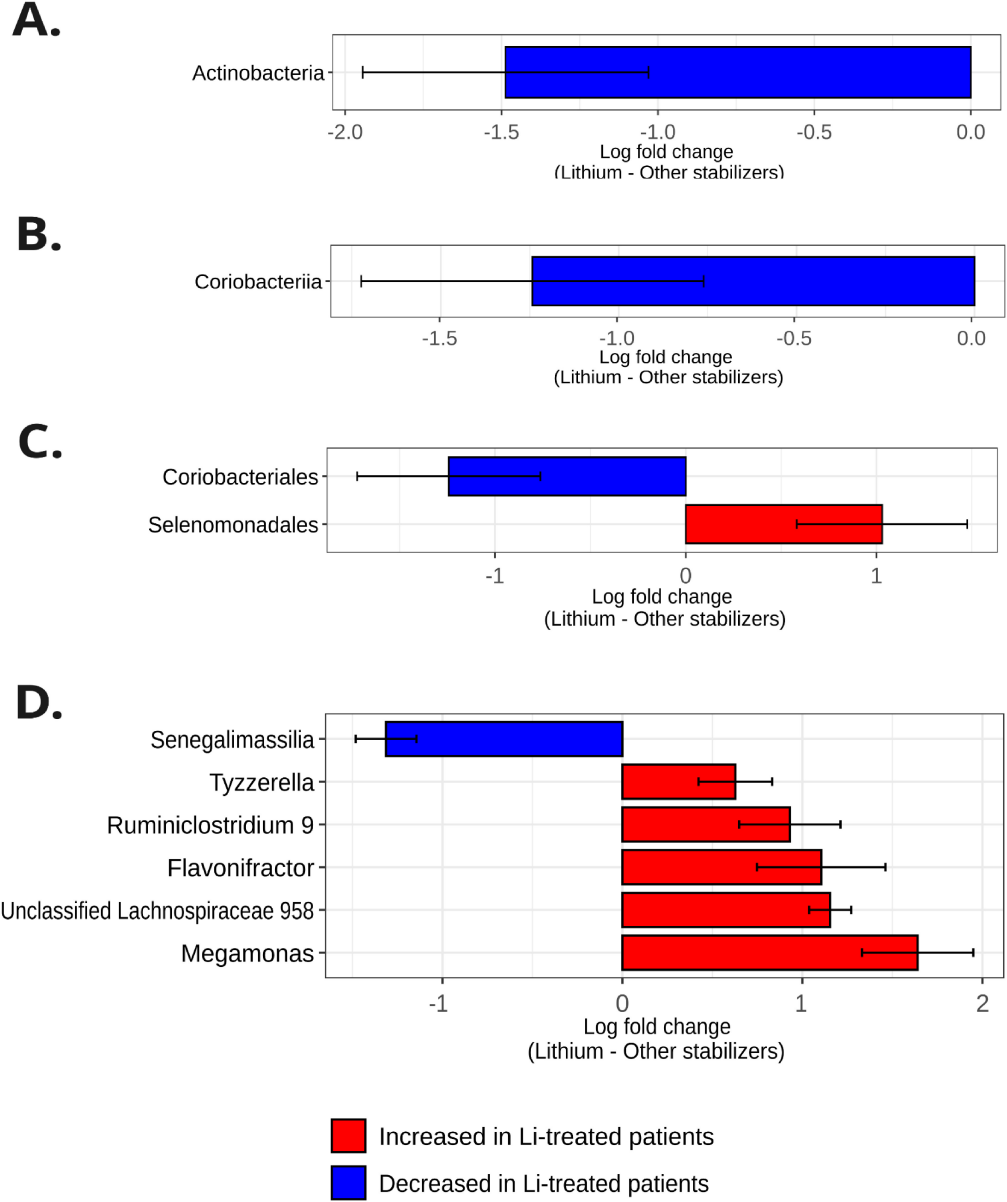
Differentially abundant taxa analysis between patients treated with Li and patients treated with other mood stabilizers. Taxa with significant changes in abundance are shown as log fold changes (LFC) with other mood stabilizers as the reference group. *P*-values were corrected for multiple testing using the Benjamini-Hochberg method. Only taxa with adjusted *p*-value < 0.05 and passing the sensitivity analysis of ANCOM-BC2 are shown. Error bars represent standard error. Each panel refers to a different taxonomic level analysis. **(A)** Phylum. **(B)** Class. **(C)** Order. **(D)** Genus. No significant taxa at the family level were found with differential abundance. Red bars represent taxa that are differentially abundant in Li-treated patients compared with patients treated with other mood stabilizers; blue bars indicate taxa that are relatively depleted in the same patients. Covariates in the model included: age, years of illness, number of manic episodes, physical activity and hypertension, with treatment as the main variable for comparison. For each taxon, the corresponding FDR-adjusted q-values are as follows: Actinobacteria (*q* = 0.0073); Coriobacteriia (*q* = 0.0334); Coriobacteriales (*q* = 0.0286); Senegalimassilia (*q* = 0.000028); Selenomonadales (*q* = 0.0457); *Megamonas* (*q* = 0.0152); *Tyzzerella* (*q* = 0.0341); *Ruminiclostridium 9* (*q* = 0.0088); *Flavonifractor* (*q* = 0.0152); *Unclassified Lachnospiraceae 958* (*q* = 0.0009). Taxonomic annotations follow SILVA v132 nomenclature; updated ICNP-compliant phylum names are provided as follows: Actinobacteria (Actinomycetota).

Differential abundance analysis at different taxonomic levels revealed several taxa with significant changes in relative abundance between patients treated with Li and those on other mood stabilizers (Figures 3A–D and Supplementary Table 5). Using the Benjamini-Hochberg method to correct for multiple testing, significant differences were observed with a *p*-value threshold of < 0.05.

In patients treated with Li, we observed a significant reduction in the relative abundance of the Actinobacteria (Actinomycetota) phylum and several taxa within it, including Coriobacteriia, Coriobacteriales, and the *Senegalimassilia* genus. Within the Firmicutes (Bacillota) phylum, we observed an increase in the Selenomonadales order and the *Megamonas.* genus, as well as the *Tyzzerella*, *Ruminiclostridium 9*, *Flavonifractor*, *Unclassified Lachnospiraceae 958*, all belonging to the Clostridia class. The analysis was controlled for potential confounders, including age, hypertension, physical activity, illness in years and number of manic episodes.

**FIGURE 4:**
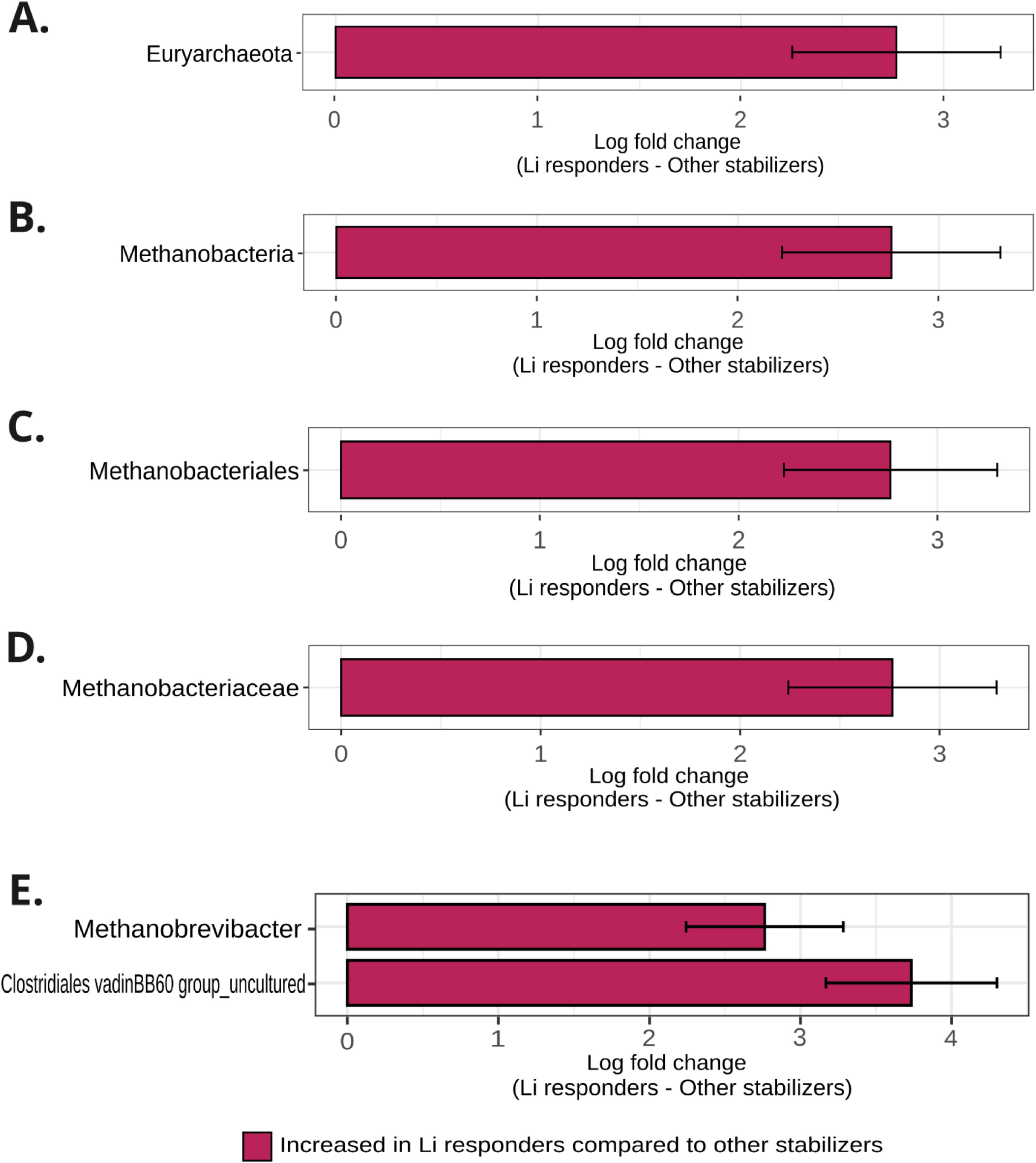
Differentially abundant taxa analysis between Li-responders and patients treated with other mood stabilizers. Taxa with significant changes in abundance are shown as log fold changes (LFC) with other mood stabilizers as the reference group. *P*-values were corrected for multiple testing using the Benjamini-Hochberg method. Only taxa with adjusted *p*-value < 0.05 and passing the sensitivity analysis of ANCOM-BC2 are shown. Error bars represent standard error. Each panel refers to a different taxonomic level analysis. **(A)** Phylum. **(B)** Class. **(C)** Order. **(D)** Family. **(E)** Genus. The bars represent taxa that are differentially abundant in Li-responders compared with patients treated with other mood stabilizers. No decreased taxa at any taxonomic level were found in Li-responders compared to other mood stabilizers. Covariates in the model included: age, years of illness, years of treatment, antipsychotic treatment, total number of episodes, physical activity and hypertension, with Li response as the main variable for comparison. No significant differences were found in taxa between Li-responders and non-responders using the covariates: use of additional mood stabilizers, antipsychotic treatment, physical activity, and MedDiet score. No significant taxa were found between Li non-responders compared to patients treated with other mood stabilizers using the covariates: years of treatment, use of additional mood stabilizers, number of manic episodes, and total number of episodes. For each taxon, the corresponding FDR-adjusted q-values are as follows: *Clostridiales vadinBB60 group_uncultured* (*q* = 0.0030); Euryarchaeota (*q* = 0.0015); Methanobacteria (*q* = 0.0057); Methanobacteriales (*q* = 0.007); Methanobacteriaceae (*q* = 0.0036); *Methanobrevibacter* (*q* = 0.0044). Taxonomic annotations follow SILVA v132 nomenclature; updated ICNP-compliant phylum names are provided as follows: Euryarchaeota (Methanobacteriota).

The same analysis based on the response to Li treatment revealed significant alterations in the GM especially in Li-responders when compared with patients treated with other mood stabilizers. In particular, we observed an increase in Li-responders in the Euryarchaeota (Methanobacteriota) phylum and in the correlated taxa Methanobacteria, Methanobacteriales, Methanobacteriaceae, and *Methanobrevibacter*, and in the *Clostridiales vadinBB60 group_uncultured* genus belonging to the Firmicutes (Bacillota) phylum. No statistically significant differences were observed in either Li responders or Li non-responders compared with patients treated with other mood stabilizers (Figures 4A–E and Supplementary Table 5).

#### 3.2.3 Functional metagenome prediction analysis

The functional analysis revealed 42 MetaCyc pathways statistically decreased in patients treated with Li compared to patients treated with other mood stabilizers (*q* < 0.05). For Li response, no statistically significant differences were found between responders and non-responders, as none of the associations exceeded the conventional significance threshold (*q* < 0.05) and all adjusted *p*-values were > 0.9. However, comparisons between Li-responders and others, as well as between Li non-responders and others, were suggestive at *q* < 0.10 (see Supplementary Figure 5 and Supplementary Table 6).

**FIGURE 5:**
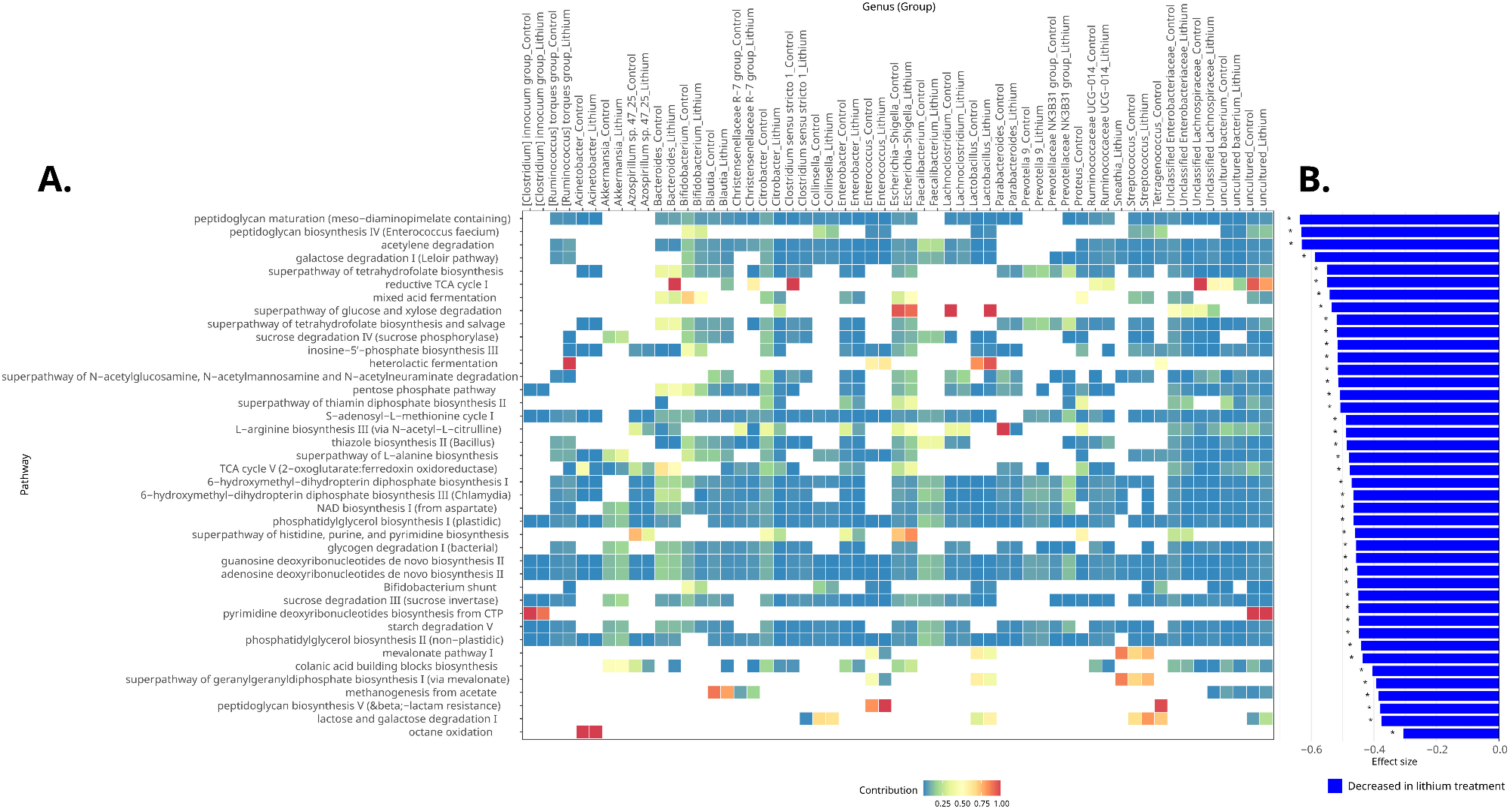
Predicted functional pathways differing between patients treated with Li and patients treated with other mood stabilizers. Predicted functional metagenomic pathways were inferred using PICRUSt2 based on the Metabolic Pathway (MetaCyc) database. Statistical comparisons of pathway abundances between groups were performed using ALDEx2 with Other mood stabilizers as the reference group. Statistical significance was evaluated with the Wilcoxon rank-sum test, and *p*-values were corrected for multiple testing using the Benjamini-Hochberg method. Only pathways with adjusted *p*-value < 0.05 are shown. **(A)** Taxonomic contribution of the 30 genera with the highest proportion to each pathway. Taxonomic contribution was calculated as the mean per group of the relative abundance of taxonomy per function and per sample (group mean of the column “norm_taxon_function_contrib” generated by PICRUSt2). **(B)** Effect size of each statistically significant differing pathway. * = adjusted *p*-value < 0.05.

The predictive functional analysis of the GM revealed a distinctive metabolic profile associated with Li treatment. In line with the observed taxonomic alterations, Li-treated patients showed a significant reduction in numerous pathways involved in bacterial structural biosynthesis, carbohydrate metabolism, primary fermentation, and the biosynthesis of nucleotides and essential cofactors compared with those treated with other mood stabilizers. Among the most markedly reduced pathways were *peptidoglycan maturation (meso-diaminopimelate containing)*, *peptidoglycan biosynthesis IV (Enterococcus faecium)*, and *peptidoglycan biosynthesis V (β-lactam resistance)*. In parallel, a reduction was observed in multiple carbohydrate degradation pathways, including *galactose degradation I (Leloir pathway)*, *sucrose degradation IV (sucrose phosphorylase)*, *sucrose degradation III (sucrose invertase)*, *lactose and galactose degradation I*, *starch degradation V*, and the *superpathway of glucose and xylose degradation*, as well as in fermentative pathways such as *mixed acid fermentation*, *heterolactic fermentation*, *acetylene degradation*, and the *Bifidobacterium shunt* (see Figure 5 and Supplementary Table 7).

## 4 Discussion

In the present study, we analyzed the GM of patients with bipolar disorder treated with Li, comparing it with that of patients receiving other mood stabilizers, with the aim of identifying microbial signatures associated with treatment exposure and clinical response. Alpha- and beta-diversity analyses did not reveal significant differences between groups, except for a reduction in Pielou’s evenness in Li-treated patients compared with those treated with other mood stabilizers. This suggests not a global depletion of the microbial ecosystem, rather a selective reorganization in which specific taxa become favored over others.

One of the most consistent findings was the significant reduction in the Actinobacteria (Actinomycetota) phylum, particularly the Coriobacteriia class, the Coriobacteriales order and the *Senegalimassilia* genus in Li-treated patients. This result is particularly interesting in light of the work by Painold et al., who reported an increase in taxa belonging to Actinobacteria and Coriobacteriia in patients with BD compared with controls (Painold et al., 2019). Actinobacteria and taxa within the Coriobacteriia class, including Eggerthellaceae and Coriobacteriaceae families, are involved in lipid metabolism, in the processing of dietary aromatic compounds and correlate with serum cholesterol levels (Clavel et al., 2014; Lahti et al., 2013; Soukup et al., 2021).

Members of the Actinobacteria phylum, particularly *Bifidobacterium* spp., are thought to contribute to primary fermentation processes by participating in the degradation of resistant starch and plant-derived polysaccharides via glycosyl hydrolases (Binda et al., 2018). It has also been shown that members of the Actinobacteria phylum, acting as primary fermenters, contribute to intestinal lactate production, which can subsequently be utilized by specific bacterial groups, known as lactate utilizers, for butyrate synthesis (McGuinness et al., 2022). Based on our results, the reduction in Actinobacteria (Actinomycetota) phylum in Li-treated patients merely suggest a decrease in microbial lactate production and could potentially indicate a protective effect of Li, consistent with pathophysiological models linking excess lactate, both intestinal and cerebral, to BD (McGuinness et al., 2022). However, this hypothesis should be investigated in future multi-omics studies to clarify the functional implications of these microbial changes.

Scientific evidence shows that genera within the Coriobacteriia class, such as *Collinsella* and *Eggerthella*, are associated with increased intestinal permeability, IL-17–mediated inflammation, unfavorable metabolic phenotypes and, in some cases, greater severity of chronic inflammatory disorders (Alexander et al., 2022; Chen et al., 2016; Gomez-Arango et al., 2018; Iqbal et al., 2025; Lambeth et al., 2015; McIntyre et al., 2021). Conversely, *Senegalimassilia* remains poorly characterized, with studies reporting contradictory associations with metabolic and inflammatory markers (Fatoba and Simpson, 2025; Luo et al., 2023; Mairinger et al., 2023; Yin et al., 2023; Zhao H. et al., 2024). Therefore, changes in *Senegalimassilia* abundance may reflect broader shifts in host metabolic organization and microbial ecosystem interactions, rather than having a direct causal effect. Overall, the reduction in Coriobacteriia taxa in Li-treated patients suggests that Li may indirectly modulate the gut environment in a direction less favorable to taxa with pro-inflammatory potential, plausibly through lithium’s known anti-inflammatory and immunomodulatory properties (Damri and Agam, 2024).

These findings align with evidence from McGuinness et al. (2022) who documented an enrichment of lactate-producing bacteria (*Lactobacillus*, *Enterococcus*, *Streptococcus*, *Bifidobacterium*) in major depressive disorder (MDD), BD and schizophrenia. Although these microorganisms can contribute to beneficial cross-feeding pathways that generates acetate/propionate/butyrate from lactate, potentially adverse effects may emerge when lactate accumulates, a phenomenon linked to multiple disorders including neurotoxicity and mitochondrial dysfunction. Moreover, increased intestinal permeability may facilitate the systemic lactate translocation, contributing to neuropsychiatric manifestations (Aguirre-Garcia et al., 2025). Elevated brain lactate has been reported in several mood disorders, including bipolar disorder (Dogan et al., 2018; Kuang et al., 2018). Within this framework, the reduction of primary fermenters in Li-treated patients merely suggest a rebalancing of fermentative metabolism.

Lithium treatment was also associated with an increase in several Firmicutes (Bacillota) taxa, including the Selenomonadales order and its *Megamonas* genus, as well as multiple taxa belonging to the Clostridia class, such as *Flavonifractor*, *Ruminiclostridium 9* (Oscillospiraceae family), *Tyzzerella* and Unclassified Lachnospiraceae 958 (Lachnospiraceae family). The Lachnospiraceae and Oscillospiraceae families are among the most relevant SCFA producers in the human gut, particularly butyrate and acetate (Flint et al., 2012; Koh et al., 2016; Louis and Flint, 2017), with key roles in epithelial barrier integrity, immune modulation and control of neuroinflammatory processes mediated through the gut-brain axis (Kim, 2021; Ney et al., 2023; Zhao et al., 2018). McGuinness et al. also described consistent reductions in major butyrate producers in psychiatric disorders (McGuinness et al., 2022). Thus, the enrichment of secondary fermenters in Li-treated patients suggests a shift from primary fermentation toward secondary fermentation sustained by cross-feeding, in line with lithium’s capacity to enhance fermentative efficiency and to modulate regulatory T cells through GPR43-dependent mechanisms (Huang et al., 2022).

*Megamonas* contributes to the production of acetate, propionate and lactate, providing essential substrates for cross-feeding (Sakon et al., 2008). Its abundance has been associated with metabolic conditions, although it may decrease in hepatic steatosis and increase in response to prebiotic interventions (Yang et al., 2023; Zhang et al., 2023). *Tyzzerella* possesses the ability to produce aromatic amines from amino acids and express β-N-acetyl-hexosaminidase, involved in the synthesis of human milk oligosaccharide precursors. Although associated in some studies with unfavorable metabolic markers (Asensio et al., 2022; Sinisterra Loaiza et al., 2025; Tang et al., 2024), it has also been reported to increase in contexts characterized by improved psychological wellbeing or lower inflammatory stress (Wang et al., 2023), suggesting a context-dependent physiological role. Overall, the increase in *Megamonas* and *Tyzzerella* may reflect a reorganization of the intestinal metabolic network in Li-treated patients.

The *Flavonifractor* genus, also increased in the Li-treated group, has been reported as enriched in bipolar disorder independently of treatment (Coello et al., 2019; Coello et al., 2021), suggesting that its higher abundance may reflect a disorder-related trait shared by patients with BD rather than a Li-induced effect. Future studies including a healthy control group will be essential to better define the role of this taxon in the pathology of the disorder.

The analysis of Li-response subgroups revealed that the most significant differences emerged when comparing Li-responders with non-Li treated patients, whereas no statistically significant differences in the direct comparison between responders and non-responders to Li or between Li non-responders and patients treated with other mood stabilizers were found.

Responders showed an increase in methanogenic taxa, including the Euryarchaeota (Methanobacteriota) phylum and the correlated Methanobacteria, Methanobacteriales, Methanobacteriaceae and *Methanobrevibacter* taxa, as well as the *Clostridiales vadinBB60 group_uncultured* genus, belonging to the Firmicutes (Bacillota) phylum.

Methanogens consume hydrogen, supporting continuous fermentation cycles and increasing SCFA yield (Camara et al., 2021; Dirks et al., 2025; Wang et al., 2022), while enhancing ecosystem stability by reinforcing cooperative metabolic interactions within the microbiota. Their clinical relevance remains debated, having been linked to both pathological and healthy phenotypes (Camara et al., 2021; McGuinness et al., 2022; Turnbaugh et al., 2006); however, their presence has also been associated with intestinal ecosystem stability and functional resilience in long-lived populations (Palmas et al., 2022). Methanogenesis also favors acetate production (Fernandes et al., 2000), a key SCFA involved in insulin sensitivity, energy homeostasis, epithelial barrier integrity and neuroinflammation. In the present study, methanogens did not significantly differentiate responders from non-responders; therefore, no direct association with treatment response can be inferred and their specific contribution remains to be established.

The enrichment of the *Clostridiales vadinBB60* group, belonging to the class Clostridia within the Firmicutes (Bacillota) phylum, in responders compared with patients treated with other mood stabilizers is also notable. This potential SCFA-producing group (Cheng et al., 2021) has been proposed as protective in postpartum depression (Jin et al., 2024) and shown to be reduced in patients with depression compared with controls (Liu et al., 2020). It has also emerged as a potential biomarker of therapeutic response in microbiome-based interventions in murine models of Alzheimer’s disease (Menden et al., 2022). Although its functions remain incompletely defined, associations with dopaminergic/serotonergic metabolites (O’Connor et al., 2019) and resilience to colitis (Atarashi et al., 2011; Deng et al., 2022) have been described. In this study, the *Clostridiales vadinBB60* group did not show significant differences between responders and non-responders; therefore, its potential role remains to be clarified and warrants further investigation.

Functional prediction analysis confirmed that Li was associated with a reduction in primary biosynthetic and fermentative pathways, including peptidoglycan pathways, nucleotide and cofactor biosynthesis, carbohydrate degradation pathways and primary fermentative pathways such as mixed acid fermentation, heterolactic fermentation and Bifidobacterium shunt (Abedi and Hashemi, 2020; Derrien et al., 2022; Vuoristo et al., 2015). This pattern is coherent with the observed reduction in primary fermenters such as Actinobacteria (Actinomycetota) and Coriobacteriia (Binda et al., 2018) and supports the hypothesis of a shift toward secondary fermentation sustained by SCFA-producing taxa, such as Lachnospiraceae and Oscillospiraceae (Culp and Goodman, 2023).

In line with alterations in gut microbial metabolic potential observed in Li-treated patients, peripheral metabolomic profiling reported by Piras et al. (2025) in the same cohort showed treatment-associated changes in systemic energy metabolism. In particular, authors observed higher systemic levels of glucose, pyruvate, lactate, glutamine and α-ketoglutarate, along with decreased glutamate, metabolites previously implicated in bipolar disorder and modulated by Li exposure.

While our microbiome analysis indicated a reduction in primary carbohydrate fermentative capacity and a shift toward taxa associated with cross-feeding and secondary fermentation, these microbial metabolic tendencies reflect changes in intestinal substrate processing rather than direct contributors to systemic metabolite pools. At the same time, the plasma metabolomic profile described by Piras et al. suggests a rearrangement of host energy metabolism characterized by elevated circulating glucose, pyruvate and lactate, consistent with increased glycolytic flux and a relative down-regulation of mitochondrial oxidative pathways in Li-treated patients.

Taken together, the microbial and metabolomic findings may represent complementary but independent signatures of lithium exposure at different biological levels, warranting future multi-omics studies with paired data to determine whether they reflect coordinated host-microbiota metabolic interactions.

## 5 Conclusion and future perspectives

Overall, our findings indicate that Li treatment is associated with a selective reorganization of the intestinal microbiota in patients with bipolar disorder, characterized by a reduction in primary fermenters and an enrichment of taxa involved in secondary fermentation and SCFA production. Functional predictions further support this ecological shift, showing a decrease in biosynthetic and primary fermentative pathways.

The identification of taxa enriched in lithium responders, including *Methanobrevibacter* and the *Clostridiales vadinBB60* group, was derived from comparisons with patients treated with other mood stabilizers rather than from direct analyses between responders and non-responders. For this reason, no direct association with treatment response can be inferred, and their specific contribution remains to be established.

Given the observational and cross-sectional nature of the study, causal inferences cannot be established. Future research should adopt longitudinal designs including pre- and post-treatment assessments and integrate multi-omic approaches, such as fecal metabolomics, microbial transcriptomics, and targeted quantification of SCFAs and lactate, to clarify the mechanistic links between specific microbial configurations and therapeutic response. Furthermore, interventional studies are warranted to determine whether targeted modulation of the GM, through dietary strategies, prebiotics, probiotics or microbiota-directed therapeutics, may enhance Li tolerability or efficacy.

Exploring this host-microbiota interface may open new avenues for precision medicine approaches in the treatment of bipolar disorder, integrating psychopharmacology with microbiome-informed strategies.

## 6 Limitations of the study

This study presents several limitations that should be taken into account when interpreting the results. A major limitation is the absence of an untreated clinical control group, which would have enabled a clearer distinction between microbiota features associated with bipolar disorder and those attributable to pharmacological treatments. Without a reference control, it remains uncertain whether some of the observed taxonomic patterns represent disorder-specific traits or treatment-related modifications.

Furthermore, the cross-sectional design prevents establishing causal relationships between microbial composition, predicted metabolic functions and clinical response.

An additional limitation concerns the clinical heterogeneity between treatment groups. Although the covariates were included in the adjusted ANCOM-BC2 models and several microbiota-related confounders were controlled through exclusion criteria, residual confounding cannot be fully excluded.

Moreover, although the cohort was clinically well characterized, the sample size was relatively small, particularly for stratified comparisons between responders and non-responders, reducing statistical power and potentially masking subtler associations.

Finally, functional prediction obtained with PICRUSt2 rely on inferred gene content and do not necessarily reflect real microbial activity. Although useful for generating hypotheses, these predictions require validation with direct functional analyses such as metatranscriptomics, metabolomics, or targeted SCFA quantification.

## Data Availability

The datasets presented in this study can be found in online repositories. The names of the repository/repositories and accession number(s) can be found at: https://www.ebi.ac.uk/ena, PRJEB108658.
