# Supplementary material for: Gut microbiota signatures associated with lithium treatment and clinical response in patients with bipolar disorder
Source: Front Microbiol. 2026 Jun 29;17:1839847. doi: 10.3389/fmicb.2026.1839847 (PMC13384019; doi:10.3389/fmicb.2026.1839847)
Supplement: Supplementary file 1 [file Supplementary_file_1.docx]

Supplementary Material


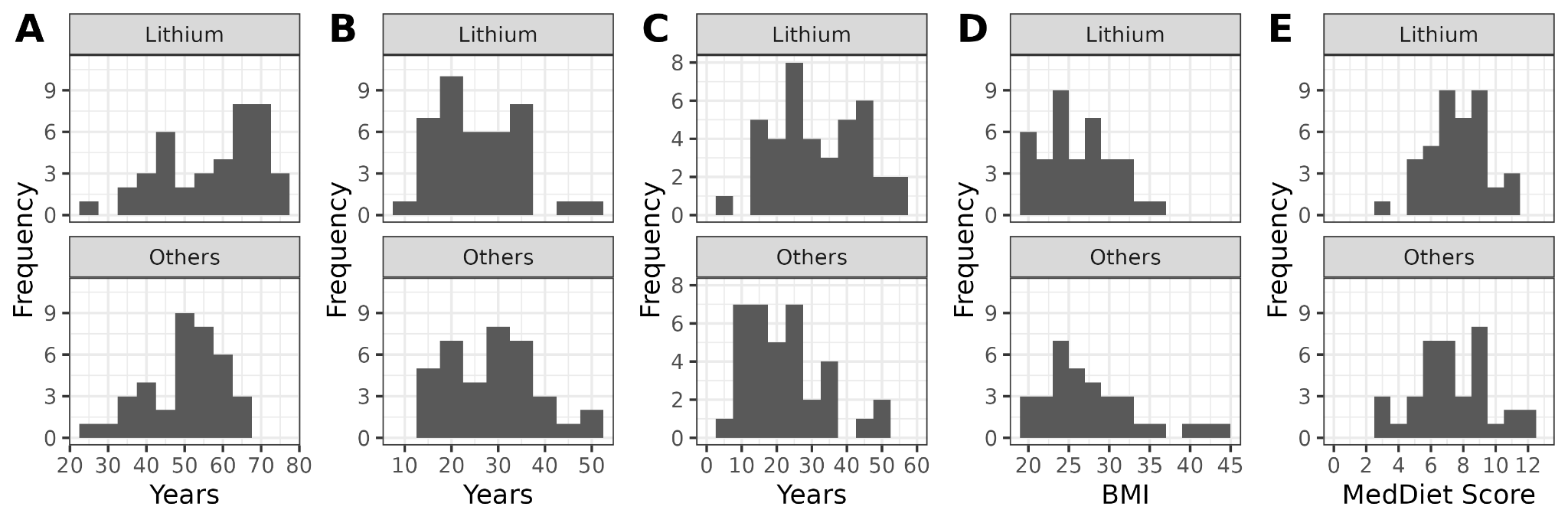


**Supplementary Figure S1.** **Histograms of clinical and demographic variables in patients with bipolar disorder, stratified by treatment.** Patients were stratified into two groups: lithium-treated patients and patients treated with other mood stabilizers. Panels show: (A) Age at sampling; (B) Age at disease onset; (C) Duration of illness (years); (D) BMI; (E) MedDietScore. Abbreviations: MedDiet = Mediterranean Diet.

**
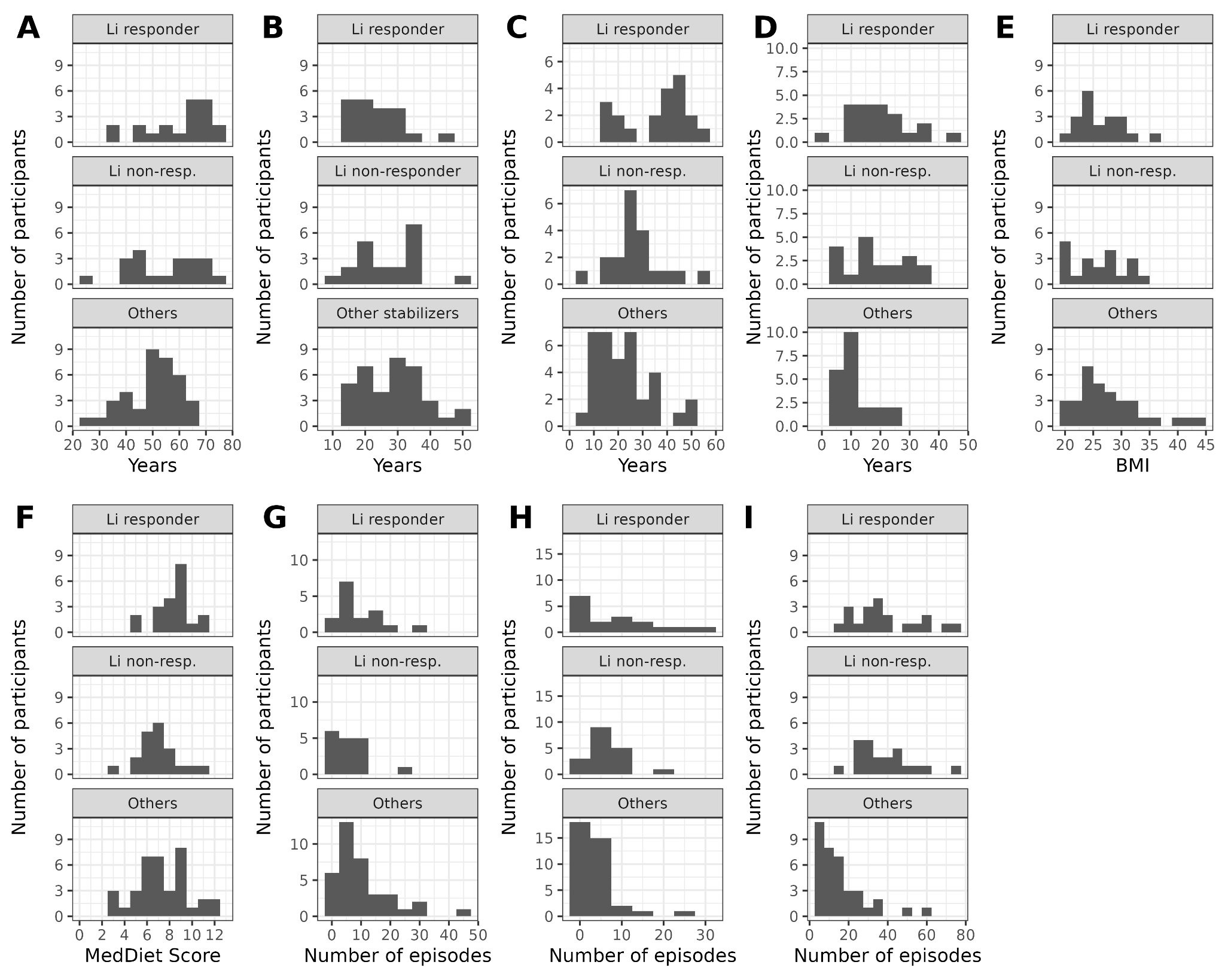
**

**Supplementary Figure S2.** **Histograms of clinical and demographic variables in patients with bipolar disorder, stratified by lithium response.** Patients were stratified into three groups: lithium responders (Li responders), lithium non-responders (Li non-responders), and patients treated with other mood stabilizers. Panels show: (A) Age at sampling; (B) Age at disease onset; (C) Duration of illness (years); (D) Duration of treatment (years); (E) Body Mass Index (BMI); (F) MedDietScore; (G) Number of depressive episodes; (H) Number of manic episodes; (I) Total number of mood episodes. Abbreviations: MedDiet = Mediterranean Diet.


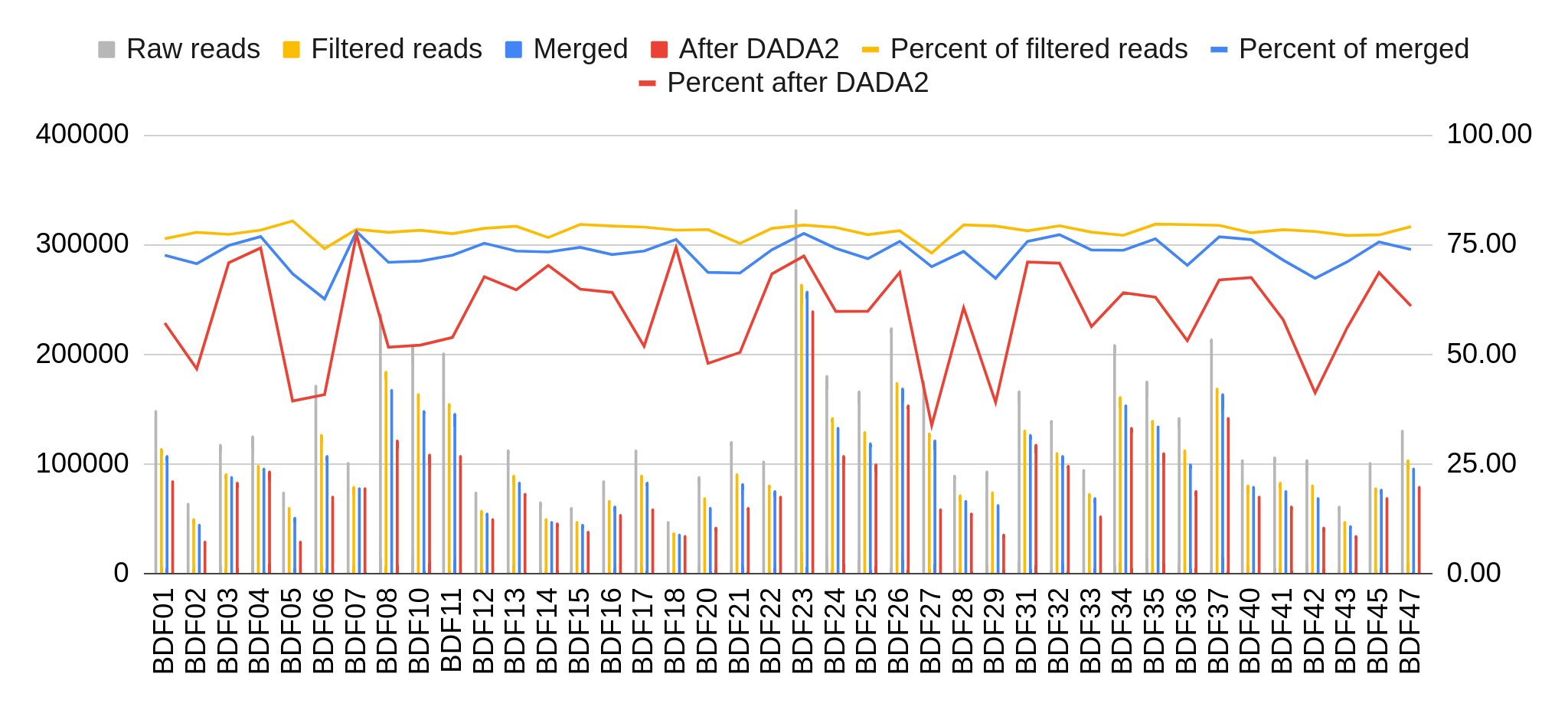


**Supplementary Figure S3.** **Sequencing reads in lithium-treated patients: absolute counts and relative proportions.** Number (left) and percentage (right) of sequencing reads obtained from samples of patients treated with lithium. The left panel shows the absolute read counts per sample, while the right panel displays the relative proportion (%) of reads per sample, allowing comparison of sequencing depth and data distribution across lithium-treated patients.


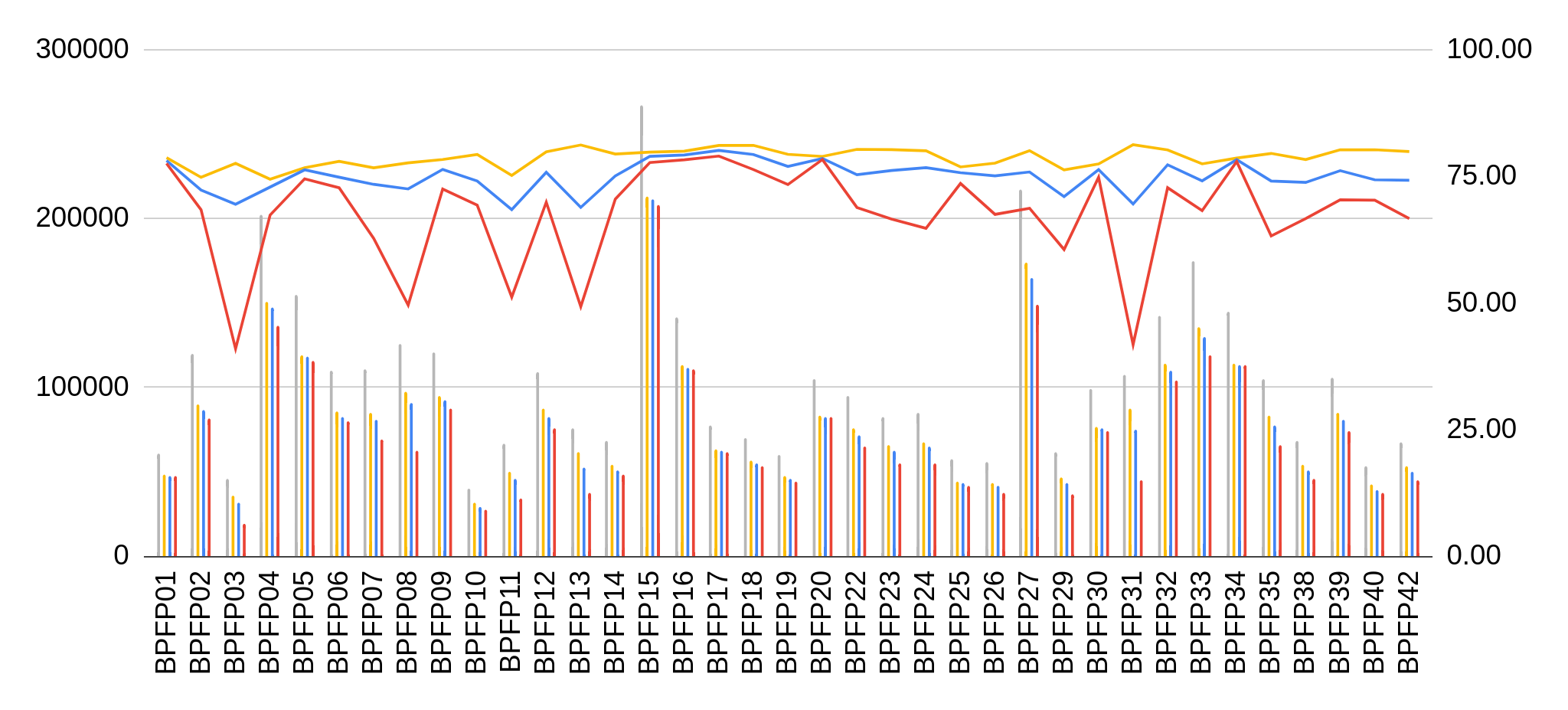


**Supplementary Figure S4.** **Sequencing reads in patients treated with other mood stabilizers: absolute counts and relative proportions.** Number (left) and percentage (right) of sequencing reads obtained from samples of patients treated with other mood stabilizers. The left panel shows the absolute read counts per sample, while the right panel displays the relative proportion (%) of reads per sample, allowing comparison of sequencing depth and data distribution across patients receiving other stabilizing treatments.

**Supplementary Table S1. Alpha diversity analysis between patients treated with Lithium and those treated with other stabilizers**

|  | **Mean (SD)** | **F-statistic / Log-likelihood** | **R2** | ***p*-value** |
| --- | --- | --- | --- | --- |
| **Observed ASVs** |  | 0.3915 | 0.03651 | 0.7213 |
| Li-treated | 227.87 (± 79.82) |  |  |  |
| Other stabilizer | 206.70 (± 76.28) |  |  |  |
|  |  |  |  |  |
| **Evenness (J: Pielou’s)** |  | 59.48 | 0.1588 | **0.0462** |
| Li-treated | 0.62 (± 0.11) |  |  |  |
| Other stabilizer | 0.67 (± 0.12) |  |  |  |
|  |  |  |  |  |
| **Simpson** |  | 84.0800 | 0.121 | 0.178 |
| Li-treated | 0.87 (± 0.12) |  |  |  |
| Other stabilizer | 0.90 (± 0.11) |  |  |  |
|  |  |  |  |  |
| **Shannon** |  | - | 0.0788 | 0.2162 |
| Li-treated | 4.78 (± 1.02) |  |  |  |
| Other stabilizer | 5.12 (± 1.04) |  |  |  |
|  |  |  |  |  |
| **Chao1** |  | 0.4734 | 0.0438 | 0.6540 |
| Li-treated | 242.42 (± 87.83) |  |  |  |
| Other stabilizer | 216.09 (± 81.43) |  |  |  |
|  |  |  |  |  |
| **Faith’s PD** |  | 0.6192 | 0.0565 | 0.538 |
| Li-treated | 17.22 (± 5.02) |  |  |  |
| Other stabilizer | 15.63 (± 4.13) |  |  |  |

Summary of alpha-diversity metrics comparing patients treated with lithium and those treated with other mood stabilizers. For each metric (Observed ASVs, Evenness (Pielou’s J), Simpson, Shannon, Chao1, and Faith’s Phylogenetic Diversity), mean and standard deviation (SD) values are reported for both groups. Statistical significance between groups was assessed using a linear regression model for metrics with normal distribution (Observed ASVs, Chao1, and Faith’s Phylogenetic Diversity), beta regression model for metrics between 0 and 1 (Evenness and Simpson), and rank-based linear model for non-normal distribution (Shannon), with relevant clinical covariates (age in years, years of illness, number of manic episodes, physical activity, and hypertension). F-statistics and R-squared for linear regression models and log-likelihood and pseudo R-squared for beta regression models are shown. A significance threshold of p ≤ 0.05 was applied.

**Supplementary Table S2. Alpha diversity analysis in Li-responders, Li non-responders, and in patients treated with other stabilizers**

|  | **Mean (SD)** | **F-statistic / Log-likelihood** | **R2** | ***Global p*-value** |
| --- | --- | --- | --- | --- |
| **Observed ASVs** |  | 0.8087 | 0.1877 | 0.6400 |
| Li responders | 237.85 (± 87.84) |  |  |  |
| Li non-responders | 221.25 (± 72.63) |  |  |  |
| Other stabilizers | 208.00 (± 77.18) |  |  |  |
|  |  |  |  |  |
| **Evenness (J: Pielou’s)** |  | 46.63 | 0.1652 | 0.2809 |
| Li responders | 0.62 (± 0.12) |  |  |  |
| Li non-responders | 0.61 (± 0.10) |  |  |  |
| Other stabilizers | 0.67 (± 0.12) |  |  |  |
|  |  |  |  |  |
| **Simpson** |  | 63.28 | 0.1427 | 0.6659 |
| Li responders | 0.88 (± 0.14) |  |  |  |
| Li non-responders | 0.87 (± 0.11) |  |  |  |
| Other stabilizers | 0.90 (± 0.11) |  |  |  |
|  |  |  |  |  |
| **Shannon** |  | 0.5054 | 0.1262 | 0.8994 |
| Li responders | 4.82 (± 1.13) |  |  |  |
| Li non-responders | 4.74 (± 0.91) |  |  |  |
| Other stabilizers | 5.12 (± 1.05) |  |  |  |
|  |  |  |  |  |
| **Chao1** |  | 0.8529 | 0.1959 | 0.5982 |
| Li responders | 255.67 (± 97.76) |  |  |  |
| Li non-responders | 232.66 (± 78.24) |  |  |  |
| Other stabilizers | 215.15 (± 82.66) |  |  |  |
|  |  |  |  |  |
| **Faith PD** |  | 0.9521 | 0.2139 | 0.5072 |
| Li responders | 18.07 (± 5.16) |  |  |  |
| Li non-responders | 16.41 (± 4.74) |  |  |  |
| Other stabilizers | 15.72 (± 4.21) |  |  |  |

Summary of alpha-diversity metrics comparing patients treated with lithium and those treated with other mood stabilizers. For each metric (Observed ASVs, Evenness (Pielou’s J), Simpson, Shannon, Chao1, and Faith’s Phylogenetic Diversity), mean and standard deviation (SD) values are reported for both groups. Statistical significance between groups was assessed using a linear regression model for metrics with normal distribution (Observed ASVs, Shannon, Chao1, and Faith’s Phylogenetic Diversity), and beta regression model for metrics between 0 and 1 (Evenness and Simpson), with relevant clinical covariates (age in years, years of illness, years of treatment, mood stabilizers other than lithium, antipsychotic treatment, number of manic episodes, total number of episodes, physical activity, MedDiet score, and hypertension). F-statistics and R-squared for linear regression models and log-likelihood and pseudo R-squared for beta regression models are shown. A significance threshold of p ≤ 0.05 was applied.

**Supplementary Table S3. PERMANOVA test on beta diversity metrics in patients treated with Li and those treated with other stabilizers**

|  | **Degrees of freedom** | **Sum of squares** | **F** | **R2** | **p-value** |
| --- | --- | --- | --- | --- | --- |
| **Aitchison** | 1 | 3295 | 1.1010 | 0.01552 | 0.157 |
| **Jaccard** | 1 | 0.4187 | 1.1013 | 0.01549 | 0.113 |
| **Bray-Curtis** | 1 | 0.5219 | 1.4082 | 0.01954 | 0.062 |
| **Unweighted UniFrac** | 1 | 0.2325 | 1.2920 | 0.01811 | 0.113 |
| **Weighted UniFrac** | 1 | 0.1833 | 1.9502 | 0.02717 | 0.074 |

The following clinical covariates were included in the PERMANOVA analysis: age in years; years of illness; number of manic episodes; physical activity; and hypertension.

**Supplementary Table S4. PERMANOVA test on beta diversity metrics in Li-responders, Li non-responders, and in patients treated with other stabilizers**

|  | **Degrees of freedom** | **Sum of squares** | **F** | **R2** | **p-value** |
| --- | --- | --- | --- | --- | --- |
| **Aitchison** | 1 | 5783 | 0.9676 | 0.03604 | 0.601 |
| **Jaccard** | 1 | 0.8055 | 1.0466 | 0.03871 | 0.194 |
| **Bray-Curtis** | 1 | 0.7361 | 0.9482 | 0.03552 | 0.561 |
| **Unweighted UniFrac** | 1 | 0.3680 | 1.0019 | 0.03682 | 0.416 |
| **Weighted UniFrac** | 1 | 0.2240 | 1.0919 | 0.04139 | 0.311 |

The following clinical covariates were included in the PERMANOVA analysis: age in years; years of illness; years of treatment; mood stabilizers other than lithium; antipsychotic treatment; number of manic episodes; total number of episodes; physical activity; MedDiet score; and hypertension.

**Supplementary Table S5. Differentially abundant taxa across treatment groups**

| **Phylum** | **Class** | **Order** | **Family** | **Genus** | **Comparison** | **Group 1 Mean** | **Group 2 Mean** | **exp(LFC)** | **Direction**  **(↓/↑)** | **p** | **q** |
| --- | --- | --- | --- | --- | --- | --- | --- | --- | --- | --- | --- |
| Actinobacteria  (Actinomycetota) | - | - | - | - | Li vs Others | 0.0214% ± 0.3965% | 0.0239% ± 0.5351% | exp(-1.4872) ≈ 77.4% decrease | **↓** | 0.0018 | 0.0073 |
| Actinobacteria  (Actinomycetota) | Coriobacteriia | - | - | - | Li vs Others | 0.0101% ± 0.1888% | 0.0137% ± 0.3003% | exp(-1.2424) ≈ 71.1% decrease | **↓** | 0.0122 | 0.0334 |
| Actinobacteria  (Actinomycetota) | Coriobacteriia | Coriobacteriales | - | - | Li vs Others | 0.0101% ± 0.1888% | 0.0137% ± 0.3003% | exp(-1.2424) ≈ 71.1% decrease | **↓** | 0.0121 | 0.0286 |
| Actinobacteria  (Actinomycetota) | Coriobacteriia | Eggerthellales | Eggerthellaceae | Senegalimassilia | Li vs Others | 0.0105% ± 0.1268% | 0.0096% ± 0.0783% | exp(-1.3133)≈ 73.1% decrease | **↓** | 0.000001 | 0.000028 |
| Firmicutes  (Bacillota) | Negativicutes | Selenomonadales | - | - | Li vs Others | 0.0425% ± 0.7527 % | 0.0538% ± 0.9431% | exp(1.0295) ≈ 2.8-fold higher | ↑ | 0.0246 | 0.0457 |
| Firmicutes  (Bacillota) | Negativicutes | Selenomonadales | Selenomonadaceae | Megamonas | Li vs Others | 0.0292% ± 0.2744% | 0.0775% ± 0.9276% | exp(1.6406) ≈ 5.16-fold higher | ↑ | 0.0032 | 0.0152 |
| Firmicutes  (Bacillota) | Clostridia | Eubacteriales | Lachnospiraceae | Tyzzerella | Li vs Others | 0.0014% ± 0.0112% | 0.0016% ± 0.0208% | exp(0.6274) ≈ 87.3% higher | ↑ | 0.0090 | 0.0341 |
| Firmicutes  (Bacillota) | Clostridia | Oscillospirales | Oscillospiraceae | Ruminiclostridium 9 | Li vs Others | 0.004% ± 0.0301% | 0.0059% ± 0.0495% | exp(0.9301) ≈ 2.54-fold higher | ↑ | 0.0016 | 0.0088 |
| Firmicutes  (Bacillota) | Clostridia | Oscillospirales | Oscillospiraceae | Flavonifractor | Li vs Others | 0.0102% ± 0.0534% | 0.0104% ± 0.0828% | exp(1.1047) ≈ 3.02-fold higher | ↑ | 0.0032 | 0.0152 |
| Firmicutes  (Bacillota) | Clostridia | Eubacteriales | Lachnospiraceae | Unclassified Lachnospiraceae 958 | Li vs Others | 0.0034% ± 0.0115% | 0.0063% ± 0.0144% | exp(1.1538) ≈ 3.17-fold higher | ↑ | 0.0001 | 0.0009 |
| Firmicutes  (Bacillota) | Clostridia | Clostridiales | Clostridiales vadinBB60 | Clostridiales vadinBB60 group_uncultured | LiR vs Others | 0.0013 %, SD: 0.0171 % | 0.0022 %, SD: 0.0308 % | exp(3.7344) ≈ 41.85-fold higher | ↑ | 2e-04 | 0.0030 |
| Euryarchaeota  (Methanobacteriota) | - | - | - | - | LiR vs Others | 0.0254 %, SD: 0.1949 % | 0.0563 %, SD: 0.3823 % | exp(2.7673) ≈ 15.91-fold higher | ↑ | 2e-04 | 0.0015 |
| Euryarchaeota  (Methanobacteriota) | Methanobacteria | - | - | - | LiR vs Others | 0.1049 %, SD: 0.468 % | 0.1002 %, SD: 0.6649 % | exp(2.7636) ≈ 15.86-fold higher | ↑ | 4e-04 | 0.0057 |
| Euryarchaeota  (Methanobacteriota) | Methanobacteria | Methanobacteriales | - | - | LiR vs Others | 0.1049 %, SD: 0.468 % | 0.1002 %, SD: 0.6649 % | exp(2.7636) ≈ 15.86-fold higher | ↑ | 3e-04 | 0.007 |
| Euryarchaeota  (Methanobacteriota) | Methanobacteria | Methanobacteriales | Methanobacteriaceae | - | LiR vs Others | 0.1049 %, SD: 0.468 % | 0.1002 %, SD: 0.6649 % | exp(2.7636) ≈ 15.86-fold higher | ↑ | 3e-04 | 0.0036 |
| Euryarchaeota  (Methanobacteriota) | Methanobacteria | Methanobacteriales | Methanobacteriaceae | Methanobrevibacter | LiR vs Others | 0.1049 %, SD: 0.468 % | 0.1002 %, SD: 0.6649 % | exp(2.7636) ≈ 15.86-fold higher | ↑ | 3e-04 | 0.0044 |

**Pairwise differentially abundant taxa analysis at the genus level in patients treated with lithium and patients treated with other mood stabilizers.** This table shows bacterial taxa with significant differences in log fold change (LFC) between the lithium-treated group (Li), the group treated with other mood stabilizers (Others), and the Lithium responders (LiR). The exponential of the LFC (exp(LFC)) represents the fold change in taxon abundance (e.g., a value of 2 indicates a doubling) of Group 1 compared to Group 2. The LFC values account for compositionality and library size differences, providing a robust measure of abundance change that is not confounded by the sum constraint of relative abundance data. For each comparison, the direction of change (↓: significantly reduced in the first group; ↑: significantly increased in the first group) is shown. The table also includes the average relative abundance (expressed as percentages) for each taxon, raw p-values (p), and adjusted p-values (q). Significance was determined using ANCOM-BC2 v.2.9.1 with a Benjamini-Hochberg correction for multiple comparisons of < 0.05.

**Table S6. Predicted functional pathways in lithium response and other stabilizers**

| **Pathway** | **Pathway category** | **MetaCyc ID** | **Comparison** | **Median CLR in Other stabilizers** | **Median CLR in Li (non-) responder** | **Median diff** | **Effect size** | **P-value** | **Adjusted p-value** |
| --- | --- | --- | --- | --- | --- | --- | --- | --- | --- |
| inosine-5'-phosphate biosynthesis III | Nucleoside and Nucleotide Biosynthesis | PWY-7234 | LiR vs Others | 3.2808 | 2.6823 | -0.7785 | -0.5208 | 0.0002 | 0.0790 |
| peptidoglycan maturation (meso-diaminopimelate containing) | Cell Structure Biosynthesis | PWY0-1586 | LiN vs Others | 4.9549 | 4.2736 | -0.6392 | -0.4480 | 0.0002 | 0.0582 |
| superpathway of L-alanine biosynthesis | Amino Acid Biosynthesis | PWY0-1061 | LiN vs Others | 4.1102 | 3.7717 | -0.7796 | -0.4291 | 0.0007 | 0.0764 |
| acetylene degradation | Fermentation | P161-PWY | LiN vs Others | 5.1816 | 4.6694 | -0.5490 | -0.4271 | 0.0003 | 0.0586 |
| peptidoglycan biosynthesis IV (Enterococcus faecium) | Cell Structure Biosynthesis | PWY-6471 | LiN vs Others | 4.7336 | 3.8938 | -0.5856 | -0.4233 | 0.0008 | 0.0770 |
| heterolactic fermentation | Fermentation | P122-PWY | LiN vs Others | 3.1334 | 2.4745 | -0.6036 | -0.3886 | 0.0016 | 0.0984 |
| mixed acid fermentation | Fermentation | FERMENTATION-PWY | LiN vs Others | 4.9659 | 4.3962 | -0.4796 | -0.3792 | 0.0015 | 0.0968 |

LiR = Lithium responders. LiN = Lithium non-responders. Others = Other mood stabilizer. Predicted functional metagenomic pathways were inferred using PICRUSt2 and classified according to the Metabolic Pathway (MetaCyc) database. Statistical comparisons of pathway abundances between groups were performed using ALDEx2 (ANOVA-Like Differential Expression). The statistical significance was evaluated using the generalized linear model (glm) function to compare multiple groups, with “Other stabilizers” as the reference group. p-values were corrected for multiple testing using the Benjamini-Hochberg method. Pathways shown had suggestive adjusted p-values < 0.1. No significant differences were found between lithium responders and lithium non-responders (all adjusted p-values were > 0.9).


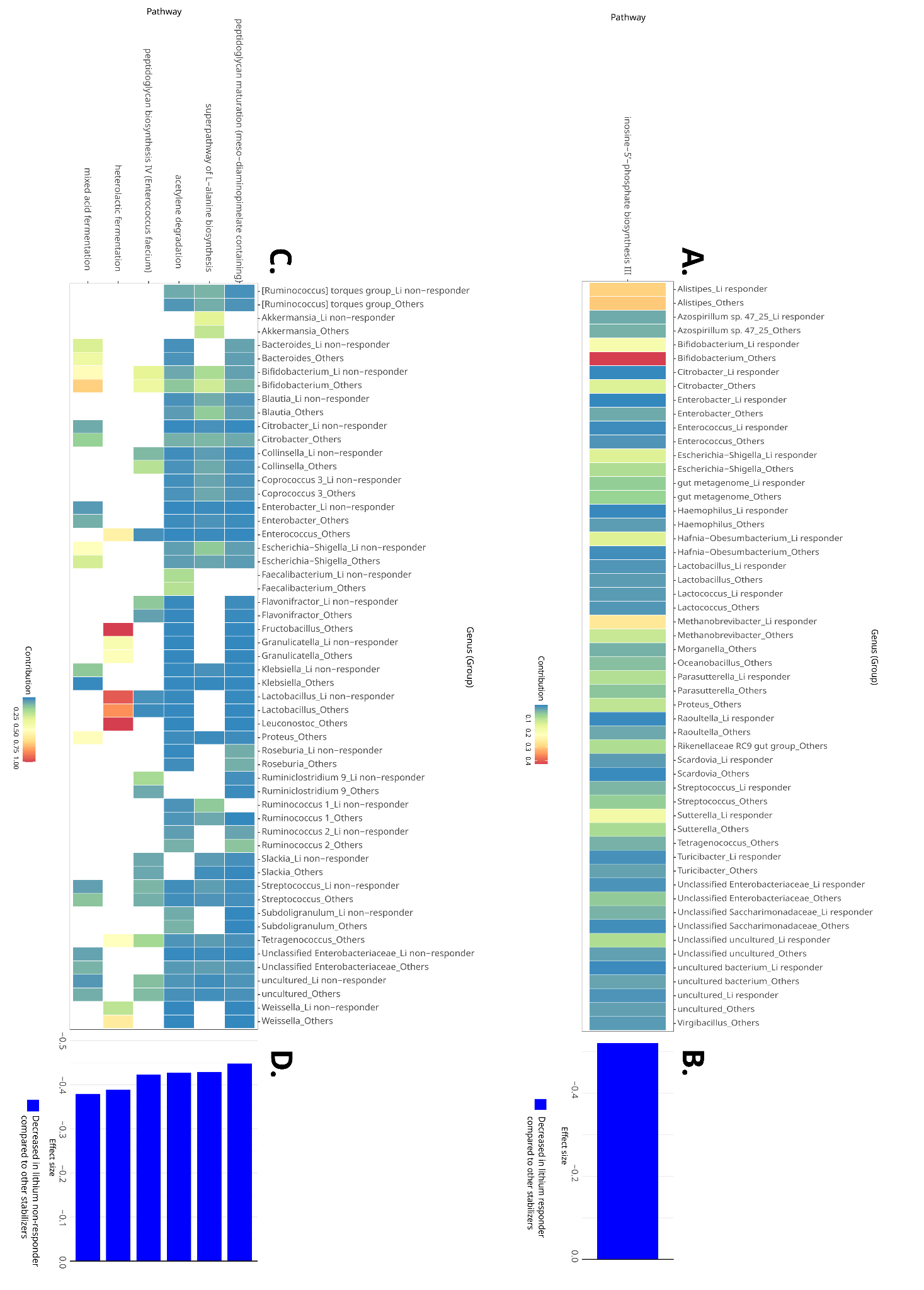


**Supplementary Figure S5. Predicted functional pathways differing between Li response and other mood stabilizers.** Predicted functional metagenomic pathways were inferred using PICRUSt2 based on the Metabolic Pathway (MetaCyc) database. Statistical comparisons of pathway abundances across multiple groups were performed using ALDEx2 with Other mood stabilizers as the reference group. Statistical significance was evaluated using the generalized linear model (glm) function, and p-values were corrected for multiple testing using the Benjamini-Hochberg method. Pathways shown in the figure had suggestive adjusted p-values < 0.1. Panel A and C: Taxonomic contribution of the 30 genera with the highest proportion to each pathway. Taxonomic contribution was calculated as the mean per group of the relative abundance of taxonomy per function and per sample (group mean of the column “norm_taxon_function_contrib” generated by PICRUSt2). Panel B and D: Effect size of each MetaCyc pathway. Panel A and B: Comparison of Li-responders vs patients treated with other mood stabilizers. Panel C and D: Comparison of Li non-responders vs patients treated with other mood stabilizers. No significant differences were found between Li-responders and Li non-responders, as all p-values were greater than 0.9 after correction.

**Supplementary Table S7. Predicted functional metagenomic pathways in BD treatment groups**

| **Pathway** | **Pathway category** | **MetaCyc ID** | **Median CLR in Other stabilizers** | **Median CLR in Li-treated** | **Median diff** | **Effect size** | **P-value** | **Adjusted p-value** |
| --- | --- | --- | --- | --- | --- | --- | --- | --- |
| peptidoglycan maturation (meso-diaminopimelate containing) | Cell Structure Biosynthesis | PWY0-1586 | 5.2746 | 4.4640 | -0.8149 | -0.6362 | 0.0001 | 0.0144 |
| peptidoglycan biosynthesis IV (Enterococcus faecium) | Cell Structure Biosynthesis | PWY-6471 | 5.0163 | 4.0983 | -0.8859 | -0.6320 | 0.0001 | 0.0151 |
| acetylene degradation | Fermentation | P161-PWY | 5.4605 | 4.8126 | -0.7259 | -0.6305 | 0.0002 | 0.0179 |
| galactose degradation I (Leloir pathway) | Carbohydrate Degradation | PWY-6317 | 5.6366 | 4.9501 | -0.7149 | -0.5887 | 0.0003 | 0.0193 |
| superpathway of tetrahydrofolate biosynthesis | Cofactor, Prosthetic Group, Electron Carrier, and Vitamin Biosynthesis | PWY-6612 | 5.1804 | 4.5309 | -0.6034 | -0.5503 | 0.0008 | 0.0232 |
| reductive TCA cycle I | C1 Compound Utilization and Assimilation | P23-PWY | 3.4421 | 2.4723 | -1.0430 | -0.5503 | 0.0003 | 0.0206 |
| mixed acid fermentation | Fermentation | FERMENTATION-PWY | 5.0915 | 4.4104 | -0.6150 | -0.5414 | 0.0004 | 0.0213 |
| superpathway of glucose and xylose degradation | Carbohydrate Degradation | PWY-6901 | 4.5505 | 3.7066 | -0.7962 | -0.5346 | 0.0010 | 0.0239 |
| superpathway of tetrahydrofolate biosynthesis and salvage | Cofactor, Prosthetic Group, Electron Carrier, and Vitamin Biosynthesis | FOLSYN-PWY | 5.4040 | 4.7528 | -0.5723 | -0.5190 | 0.0014 | 0.0261 |
| sucrose degradation IV (sucrose phosphorylase) | Carbohydrate Degradation | PWY-5384 | 4.7427 | 3.7067 | -0.8393 | -0.5186 | 0.0005 | 0.0219 |
| inosine-5'-phosphate biosynthesis III | Nucleoside and Nucleotide Biosynthesis | PWY-7234 | 3.3871 | 2.7673 | -0.7793 | -0.5166 | 0.0008 | 0.0229 |
| heterolactic fermentation | Fermentation | P122-PWY | 3.3557 | 2.6796 | -0.7086 | -0.5164 | 0.0006 | 0.0221 |
| superpathway of N-acetylglucosamine, N-acetylmannosamine and N-acetylneuraminate degradation | Amine and Polyamine Degradation | GLCMANNANAUT-PWY | 4.0561 | 3.3971 | -0.7527 | -0.5151 | 0.0006 | 0.0224 |
| pentose phosphate pathway | Pentose Phosphate Pathways | PENTOSE-P-PWY | 4.1282 | 3.2673 | -0.8404 | -0.5140 | 0.0011 | 0.0241 |
| superpathway of thiamin diphosphate biosynthesis II | Cofactor, Prosthetic Group, Electron Carrier, and Vitamin Biosynthesis | PWY-6895 | 4.4443 | 3.8765 | -0.5351 | -0.5082 | 0.0007 | 0.0225 |
| S-adenosyl-L-methionine cycle I | Amino Acid Biosynthesis | PWY-6151 | 5.2898 | 4.6425 | -0.7042 | -0.5073 | 0.0011 | 0.0244 |
| L-arginine biosynthesis III (via N-acetyl-L-citrulline) | Amino Acid Biosynthesis | PWY-5154 | 4.2626 | 3.5711 | -0.6657 | -0.4897 | 0.0018 | 0.0281 |
| thiazole biosynthesis II (Bacillus) | Cofactor, Prosthetic Group, Electron Carrier, and Vitamin Biosynthesis | PWY-6891 | 3.8351 | 3.0345 | -0.7711 | -0.4886 | 0.0009 | 0.0233 |
| superpathway of L-alanine biosynthesis | Amino Acid Biosynthesis | PWY0-1061 | 4.7545 | 3.7622 | -0.8299 | -0.4875 | 0.0013 | 0.0254 |
| TCA cycle V (2-oxoglutarate:ferredoxin oxidoreductase) | TCA cycle | PWY-6969 | 5.1163 | 4.5339 | -0.5681 | -0.4801 | 0.0024 | 0.0315 |
| 6-hydroxymethyl-dihydropterin diphosphate biosynthesis I | Cofactor, Prosthetic Group, Electron Carrier, and Vitamin Biosynthesis | PWY-6147 | 5.0093 | 4.3898 | -0.5905 | -0.4770 | 0.0021 | 0.0300 |
| 6-hydroxymethyl-dihydropterin diphosphate biosynthesis III (Chlamydia) | Cofactor, Prosthetic Group, Electron Carrier, and Vitamin Biosynthesis | PWY-7539 | 4.9690 | 4.4193 | -0.5741 | -0.4710 | 0.0029 | 0.0337 |
| NAD biosynthesis I (from aspartate) | Cofactor, Prosthetic Group, Electron Carrier, and Vitamin Biosynthesis | PYRIDNUCSYN-PWY | 5.4975 | 4.9317 | -0.5377 | -0.4658 | 0.0035 | 0.0371 |
| phosphatidylglycerol biosynthesis I (plastidic) | Fatty Acid and Lipid Biosynthesis | PWY4FS-7 | 5.9134 | 5.4172 | -0.5144 | -0.4657 | 0.0025 | 0.0318 |
| superpathway of histidine, purine, and pyrimidine biosynthesis | superpathway of histidine, purine, and pyrimidine biosynthesis | PRPP-PWY | 4.5961 | 3.8962 | -0.5200 | -0.4656 | 0.0049 | 0.0445 |
| glycogen degradation I (bacterial) | Polymeric Compound Degradation | GLYCOCAT-PWY | 6.2378 | 5.5293 | -0.5492 | -0.4601 | 0.0052 | 0.0464 |
| guanosine deoxyribonucleotides de novo biosynthesis II | Nucleoside and Nucleotide Biosynthesis | PWY-7222 | 5.7564 | 5.1920 | -0.4811 | -0.4568 | 0.0042 | 0.0411 |
| adenosine deoxyribonucleotides de novo biosynthesis II | Nucleoside and Nucleotide Biosynthesis | PWY-7220 | 5.7558 | 5.1916 | -0.4846 | -0.4568 | 0.0043 | 0.0414 |
| Bifidobacterium shunt | Fermentation | P124-PWY | 4.0054 | 3.3817 | -0.6939 | -0.4538 | 0.0023 | 0.0311 |
| sucrose degradation III (sucrose invertase) | Carbohydrate Degradation | PWY-621 | 5.3151 | 4.7484 | -0.6153 | -0.4527 | 0.0020 | 0.0290 |
| pyrimidine deoxyribonucleotides biosynthesis from CTP | Nucleoside and Nucleotide Biosynthesis | PWY-7210 | 1.7827 | 0.2338 | -1.1854 | -0.4497 | 0.0015 | 0.0264 |
| pyrimidine deoxyribonucleotides de novo biosynthesis IV | Nucleoside and Nucleotide Biosynthesis | PWY-7198 | 1.3813 | -0.2461 | -1.2086 | -0.4494 | 0.0016 | 0.0272 |
| starch degradation V | Polymeric Compound Degradation | PWY-6737 | 6.2226 | 5.5319 | -0.5370 | -0.4488 | 0.0044 | 0.0419 |
| phosphatidylglycerol biosynthesis II (non-plastidic) | Fatty Acid and Lipid Biosynthesis | PWY4FS-8 | 5.9122 | 5.4155 | -0.4888 | -0.4482 | 0.0025 | 0.0318 |
| mevalonate pathway I | Secondary Metabolite Biosynthesis | PWY-922 | -1.7868 | -3.7222 | -1.8716 | -0.4417 | 0.0026 | 0.0324 |
| colanic acid building blocks biosynthesis | Carbohydrate Biosynthesis | COLANSYN-PWY | 4.7803 | 4.2281 | -0.5458 | -0.4367 | 0.0031 | 0.0350 |
| superpathway of geranylgeranyldiphosphate biosynthesis I (via mevalonate) | Secondary Metabolite Biosynthesis | PWY-5910 | -1.2771 | -3.2032 | -1.9659 | -0.4049 | 0.0026 | 0.0324 |
| methanogenesis from acetate | Respiration | METH-ACETATE-PWY | 2.2350 | 0.8442 | -1.0935 | -0.3934 | 0.0060 | 0.0495 |
| palmitate biosynthesis II (bacteria and plants) | Fatty Acid and Lipid Biosynthesis | PWY-5971 | 3.9876 | 3.2476 | -0.8223 | -0.3858 | 0.0041 | 0.0403 |
| peptidoglycan biosynthesis V (&beta;-lactam resistance) | Cell Structure Biosynthesis | PWY-6470 | 0.2535 | -2.3214 | -2.2022 | -0.3796 | 0.0031 | 0.0350 |
| lactose and galactose degradation I | Carbohydrate Degradation | LACTOSECAT-PWY | -1.1520 | -3.7095 | -2.1779 | -0.3752 | 0.0014 | 0.0259 |
| octane oxidation | Degradation/Utilization/Assimilation - Other | P221-PWY | -1.8168 | -3.5353 | -1.9236 | -0.3052 | 0.0055 | 0.0467 |

Predicted functional metagenomic pathways were inferred using PICRUSt2 and classified according to the Metabolic Pathway (MetaCyc) database. Statistical comparisons of pathway abundances between groups were performed using ALDEx2 (ANOVA-Like Differential Expression). The statistical significance was evaluated with the Wilcoxon rank test, and p-values were corrected for multiple testing using the Benjamini-Hochberg method.

The table shows the median of the centered log-ratio (CLR) of each pathway in the two groups of comparison, the median difference in CLR values between the lithium-treated participants and the participants treated with other stabilizers (Median diff.), the median effect sizes (Median diff. / median of the largest difference in CLR values within the Li-treated group and other stabilizers group), p-values, and q-values. Negative effect sizes indicate a decrease in the Lithium-treated group compared to Other stabilizers; positive effect sizes would indicate an increase.
